# The Meaning of Leadership in Medical Education in the Pan American Health Organization Member States: A Stakeholder Analysis and Interviews

**DOI:** 10.3389/ijph.2026.1608502

**Published:** 2026-02-26

**Authors:** Pablo Rodríguez-Feria, Natalia Giraldo-Noack, Susana Garcia-Arango, Martina Paric, Suzanne Babich, Laura Magaña, Luis Jorge Hernández-Flórez, Katarzyna Czabanowska

**Affiliations:** 1 Department of International Health, Care and Public Health Research Institute (CAPHRI), FHML, Maastricht University, WHO CC for Public Health Leadership and Workforce Development, Maastricht, Netherlands; 2 Departamento de Salud Pública, Facultad de Medicina, Universidad de los Andes, Bogota, Colombia; 3 Hertie School of Governance, Berlin, Germany; 4 Psychology Department, University of the Andes, Bogota, Colombia; 5 Department of Community and Global Health, Richard M. Fairbanks School of Public Health, Indiana University Indianapolis, Indianapolis, IN, United States; 6 Association of Schools and Programs of Public Health (ASPPH), Washington, DC, United States; 7 Program in Public Health, Schools of Medicine and Government, Universidad de Los Andes, Bogota, Colombia; 8 Department of Health Policy Management, Institute of Public Health, Faculty of Health Sciences, Jagiellonian University, Kraków, Poland

**Keywords:** education, medical, undergraduate, leadership, interview, stakeholder analysis, americas

## Abstract

**Objective:**

Conceptualizing leadership in undergraduate medical education in the member states of the Pan American Health Organization.

**Methods:**

Semi-structured interviews were done with the stakeholders who worked in a member state of the Pan American Health Organization. Three steps were followed to identify them: stakeholder analysis, networking by the authors, and snowballing. Content analysis and a member checking process were used to achieve agreement on the themes and codes.

**Results:**

Thirty-four stakeholders were interviewed. *Health-promoting leadership* and *expanding the borders of medicine* are the central concepts for education and training in leadership as they focus on achieving people’s wellbeing and health. *Leading and leadership antonyms, models of our own: Leadership signature in the Americas, and challenges: health, public health*, *and health systems and services* are the peripheral domains that aim to differentiate leadership from other concepts and the target audience, which includes undergraduate medical education, other professions, and individuals without a profession.

**Conclusion:**

We encourage the member states of the Pan American Health Organization to consider this research as foundation for leadership education and training which can contribute to strengthening capacities in undergraduate medical education and other audiences to enhance population wellbeing and health across the Americas.

## Introduction

The importance of leadership education and training for the health workforce and other professionals working in health and the health systems and services has been well documented (Supplementary Material 1) [1–4]. Consequently, organizations with a health and/or education mission have been teaching leadership across the health workforce and other professions [5–28].

A critical pathway has been established for leadership education and training in medicine, other professions and in the general population (people) in the Pan American Health Organization member states [14]. To follow it properly, it is necessary to understand what leadership is and how it relates to the health, public health and health systems and services challenges that can be addressed through leadership education and training. Additionally, comprehending leadership allows for directing its teaching to a target audience and creating leadership competency frameworks to support education and training.

However, leadership faces the challenge of having multiple definitions, and the critical pathway requires a conceptualization of leadership by multiple stakeholders involved in leadership education and training [14]. Three literature reviews have aimed to conceptualize leadership for health and health systems and services. Puertas et al. conducted a review in English and Spanish, identifying a definition of leadership in primary care in Mexico [1]. Fennell studied English literature and found 11 definitions of health-related leadership [3]. Rodriguez-Feria et al. searched literature in Spanish, English, and Portuguese, identifying 23 definitions written in English for leadership education and training in undergraduate medical education (UME) with an interprofessional and trans-professional education approach [12].

Moreover, medical education serves a vital role in preparing the health workforce to meet evolving patient care demands and lead transformative changes within the health systems and services. Beyond imparting knowledge, educators play a pivotal role in inspiring learners to make meaningful contributions to healthcare delivery. Effective leadership in medical education involves not only driving curriculum development and educational practices but also leveraging vision, strategy, and influence to navigate challenges and align initiatives with organizational goals [29].

Evidence-based leadership development underscores the importance of evidence-driven strategies integrating didactic learning, experiential activities, mentorship and feedback to foster competencies like emotional intelligence and teamwork [30]. Leadership competency frameworks outline essential competencies such as strategic vision and ethical judgment, preparing the future health workforce for complex environments [31]. Lastly, interprofessional and trans-professional education leadership approach stresses collaborative competencies across disciplines, crucial for improving patient outcomes and healthcare delivery [32].

Four reviews about leadership competency for UME have been published by Matsas et al, Webb et al, James et al, and Rodríguez-Feria et al between 2014 and 2023 [9–12]. Although Latin America and the Caribbean are important regions globally, possessing a significant 23% of the world’s medical schools (776 out of 3384 worldwide as of 2018 and surpassing Europe with 479 medical schools), these reviews have not focused on the experiences from these regions [4].

Some qualitative UME leadership studies include those of Keijser et al, Rajeh et al and Gordon et al who have all sought to conceptualize leadership through semi-structured interviews with UME and other audiences, these have also not focused on these specific regions [26–28]. Although there are also leadership studies in UME in Canada and the United States of America [9–11], there is no systematic research on understanding leadership in the Pan American Health Organization member states. Therefore, the aim of this research was to explore how leadership is conceptualized by stakeholders interested in leadership education and training in UME in the Pan American Health Organization member states.

## Methods

This project compromised two phases: a stakeholder analysis (the guide) [33] and stakeholder interviews. Standards for reporting qualitative research were used (Supplementary Material 2) [34].

### Sampling

Stakeholders were selected based on specific criteria: current employment within Pan American Health Organization member states and demonstrated interest in teaching leadership in UME, as evidenced by their published research. The Public Health Leadership Competency Framework Model guided the exploration of leadership expertise or interest, encompassing eight domains, including political leadership [35]. Stakeholders were eligible if they covered at least one of these domains. Participation required proficiency in either English or Spanish (Supplementary Material 3).

Non-probabilistic purposive sampling was used. Three methods for stakeholder identification were implemented: A literature review (sampling 1-S1-), authors’ professional network (sampling 2 -S2-), and a snowball sampling technique (sampling 3 -S3-) was utilized during the interviews, where stakeholders were asked for recommendations based on eligibility criteria. Identified stakeholders were contacted via email, receiving an invitation letter and informed consent.

### Interview Process and Data Collection

The guide recommended identifying and training neutral interviewers for the project [33]. Two psychology students were chosen to conduct and analyze the interviews. They did not have any personal nor professional relationship before the interviews. A draft interview scenario from the guide was used. Probe questions were initially developed in English by three authors and then translated into Spanish by one interviewer. The second interviewer cross-checked the English and Spanish versions. Pilot interviews were conducted to assess question clarity and interview flow. Semi-structured interviews were employed to explore stakeholders’ expertise, featuring open-ended questions that encouraged in-depth discussion on the research topic, fostering a natural conversational flow for the interviewees [36–38].

The research paradigm selected for this study was the constructivist paradigm. We aimed to explore how knowledge is constructed to conceptualize leadership within social environments, emphasizing the importance of understanding diverse perspectives and contextual nuances among stakeholders. Adopting a constructivist approach allowed us to delve into the subjective realities of stakeholders, gaining insights into their unique interpretations and experiences of leadership [36–38].

### Interviews

The interviews were conducted both in person in the city of Bogotá, Colombia, and online via Zoom. Each stakeholder was interviewed once. To respect privacy, the interviews were not recorded on the platform but were instead captured using an audio recorder. Each interview began with a participant introduction. The interviewers then informed the stakeholders when the recording started. And project-related questions and concerns about consent were then addressed.

After each interview, stakeholders were informed that the interview would be translated from Spanish to English or transcribed if already conducted in English. Once the English transcription was ready, it was sent to the stakeholders for approval. Upon approval, the recording was deleted. The transcriptions omitted stakeholder’s names, nationalities, and affiliations, replacing them with identifiers like “participant 1 … ∞ .” In the same email, stakeholders received a table to complete with names, surnames, affiliations, and research expertise in the field. This table was organized alphabetically by stakeholders’ surnames.

The interviews began in September 2023 and concluded upon reaching information saturation in November 2023. After conducting 34 interviews several signs indicated data saturation: no new data, no new themes and no new coding. These saturation criteria were assessed as proposed by Guest et al [39].

### Data Analysis

An inductive content analysis methodology was used, as it allowed for an open and flexible exploration of data. This approach facilitated the emergence of patterns and themes directly from the data without predefined categories, capturing the richness and complexity of the information and uncovering insights into participant’s attitudes, beliefs, and behaviors [36–38].

A pilot test of six transcriptions, which were independently coded by two separate teams using ATLAS.ti was conducted. Subsequently, the teams convened to discuss the coding process, providing guidance, comparing results, developing themes, and addressing any discrepancies. A member checking process was done by three independent authors to achieve agreement on the themes and codes identified in the pilot test sample. Two authors then coded the remaining transcriptions, and the initial three authors performed member checking on these transcriptions. Coding disagreements were addressed during online meetings with the coding team. Two authors were involved in the coding process, and their codes were then reviewed in meetings by the other authors. The transcription and translation to English of the interview were shared with every participant along with the interpretations and themes prepared by the authors after coding. This allowed the participants to contribute to the process and minimize interpretative bias by the researchers.

Each stakeholder was characterized based on the following criteria: they were labeled as “internal” (I) if affiliated with one of the institutions promoting the study, or “external” (E) if affiliated with a different organization. Language used for the interview, and stakeholders’ gender and location were also noted. Subsequently, stakeholders’ professional websites were analyzed, and queries were conducted to extract their affiliations and academic background. Affiliations included national organizations such as universities, hospitals, and associations, among others. Multilateral organizations were defined as those with legal bounds between two or more countries. Academic backgrounds were categorized as follows: graduate education, undergraduate education, and postgraduate education.

### Ethical Issues Pertaining to Human Subjects

Ethical permission was given by the ethics committee of Maastricht University with the reference: FHML-REC/2023/099. Informed consent was obtained from all interviewees, including a discussion on the anonymity of their interviews. During the analysis phase, each participant’s name was replaced with a unique identifier to ensure confidentiality and to protect their identity. The interview protocol and the transcripts can be accessed at https://osf.io/mg5zq/?view_only=5f1714713e544a6bb108075d3bd99ea0.

## Results

Most of the stakeholders, primarily from external organizations (29/34, 85%), were identified through a literature review (25/34, 74%) as the sampling method. Spanish was the predominant language used in the interviews (22/34, 65%). Interviewees represented a gender-balanced group (17/34, 50%) mainly located in the US (10/34, 29%), Mexico (7/34, 21%), and Colombia (6/34, 18%). The academic background of stakeholders included undergraduate training, master’s, doctoral, residency, or fellowship such as medicine, public administration and social communication (Supplementary Material 4).

Stakeholders were affiliated nationally, primarily with universities (30/34, 88%), while others were employed in multilateral organizations such as the Pan American Health Organization and the Andean Health Body–Hipólito Unanue Convention (2/34, 6%). Half of the stakeholders reported roles such as tutoring for the Impact Leadership Course on Leadership for Policy Management, Regulation, and Planning in the Andean Region (Supplementary Material 4).

The interviews yielded five themes detailing the conceptualization of leadership in Pan American Health Organization member states, specifically focusing on the Latin America and the Caribbean regions (Supplementary Material 5; Table 1). The conceptualization includes central and peripheral themes that highlight the importance of leadership education and training. The central themes revolve around individuals, families and communities and reflect the core mission of health and health systems and services: *health promoting leadership* and *expanding the borders of medicine*. The peripheral themes include *Leading and leadership antonyms, models of our own,* and *challenges: health, public health, and* health systems and services (Figure 1).

**TABLE 1:** Stakeholders’ quotes. (Member states of the Pan American Health Organization.2023).

#	Quote
1	Participant #2: “At least what I feel is more accessible is to make students health promoters. Teach them how to promote health. Here, for example, we have a group of public health professionals who focus on tobacco prevention, and they have a system called ABC that they easily introduce to undergraduate students. Most of our students from all disciplines have the opportunity to do rotations in primary care, and a significant part of what they are taught and experience there is related to prevention. In our case, for example, issues like tobacco prevention and oral hygiene are basic topics that we teach and promote extensively in undergraduate education. However, students encounter a completely different reality when they enter the workforce. In practice, there is often more emphasis on treating diseases than preventing them. We are constantly trying to promote a different approach as healthcare professionals, but the reality of the job market often clashes with that”
2	Participant #22: “I do think that leading self, starts with understanding self, how we see the world, what our inherent conflict preferences are, and how we make those connections, is an important piece of leadership development. To me, those capabilities can be developed even before medical school or, your psychology student, you know you can build this capacity at a young age to really understand self. I don't know that we studied that, but that's my sense is that we could start to build some of that better understanding of self. How we then engage with other individuals and actually forming true teams, as opposed to just being a group of people, building on each other's skills and supporting each other, I think are incredibly important to actually build those relationships”
3	Participant #27: “in my mind leader is a person. Leadership is about the qualities and skills of the person that allows for change to happen. It’s really about making change happen if they’re in a positive direction”
4	Participant #07: “For example, we could say it's one thing to have a boss who bosses you around and says, “you have to do this and this and this and this and this because I said so and that's the way it is”, right? And a leader who is usually someone who inspires you, who sometimes doesn't necessarily have to be saying ”do this because I say so” but people follow just because he has the condition and the skills to do it, so that's what's left. Leadership terms could be misunderstood in that aspect, to be leaders by forcing people to follow instead of generating a positive influence in the group so that the group is convinced of your message of the need to make a change”
5	Participant #30: “Well, for me, they are two sides of the same coin. They are usually contrasted, but I actively believe that they are not opposed. Leadership without management becomes ineffective leadership that never translates into practice, and management without leadership is like moving forward without advancing, without a clear direction. So, leadership creates a destination, and management builds a path to that destination. You cannot have one without the other. While we typically contrast them, I believe that is a conceptual mistake. For me, they are two indispensable components. There are managers who are not leaders, that's true. But they are part of a team where there is a shared vision articulated by him or the leaders. So, it's not so much that each individual has to be simultaneously a leader and a good manager, but that an organization, an institution, a community has to have both components. They reinforce each other”
6	Participant #23 “I think this is a matter of what I would call authenticity. Pablo, you know authentic means that you live the life that you teach. I often think of Mahatma Gandhi’s quote. My life is my message. My life is my message. And I think that universities and teachers and educators have a particular responsibility to demonstrate authentically the behaviors of their spousing for their students. That means being a great doctor being. Being a great doctor means, I think, being socially responsible as well as caring for individuals and your patients. I think that there's really no room, in my view, for hypocrisy and that regard you're describing hypocrisy among political leaders. That you know profit off of war and battles and so on. Now, you know having said that people are people and people are complex, right and you'll always have some degree of misbehavior or some”
7	Participant #30: “And this requires the leader to have a clear sense of legacy. For oneself, legacy is essential to the idea of leadership. Legacy is what one contributes to the development of an institution or organization. How one creates, adds, or contributes to an institution that exists before one arrives and will persist after one’s tenure in that institution ends. Legacy is what one leaves behind, and that is the key concept of leadership. Without a clear legacy concept, it is very difficult to activate the v, strategy, and execution plan that I mentioned before”
8	Participant #32: “I see that medical education is going through a crisis, especially in Latin America. This is primarily because, since the Flexner report in the United States and Canada, our faculties have been heavily influenced by a purely scientific perspective and the widespread use of medications generalized in the population, programs have been developed based on separate subjects and departments, and in my country, there have been attempts to modify the curriculum to transition to an integrated model, which is uncommon in Latin America. Few universities in Latin America, to my knowledge, have successfully implemented an integrated curriculum with problem-based learning. However, this has led to various administrative issues. In my faculty, the curriculum is course-based and still follows a structure of basic, preclinical, and clinical stages. I believe it’s a critical moment for Latin America to rethink the type of professionals we aim to produce. We are going through a critical stage because, even though our curriculum is designed for general practitioners in primary care, there are limited job opportunities in primary care in my country. There are very few professional growth opportunities in primary care and it’s not considered important. There is little emphasis on it, and more than 95% of our graduates aspire to specialize as the next step, which is seen as a mandatory requirement to practice medicine. This is why I talk about a critical stage – it depends a lot on what medical schools do to find the true path for the ideal training of a medical professional. This begins with defining what we want to achieve in the undergraduate degree. That is where we are facing many challenges in reality, (unadible) in Colombia, where the implementation of these changes has only started to take place after 15 years, even after its inception, only in certain isolated implementation experiences. Therefore, we also have to consider whether we are going to train a doctor for that model of family, community, intercultural health. Because currently, as I tell you, the progression of our students through our career, and once they graduate, what they mostly seek is a specialization. And that obviously is in dissonance, it doesn’t align with the profile of a primary care physician that we should supposedly be aiming for. The majority of our graduates are seeking specialization as the safest path for their professional practice and even for their personal development”.
9	Participant #21: “I think there's a lot of challenges with the US health care system. I, unfortunately, can't speak to the Colombian health care system, but from what I know about living here locally and working in the emergency department, there's a huge problem with health care disparities and health outcomes at very significantly by socio-economic factors, race, gender, and sex, ethnicity. There's major issues with social determinants of health having a big impact on health outcomes. There's issues with people being able to access and afford preventive care that leads to a lot of use of the emergency department, which, you know, contributes to the high cost of our health care system. There's issues with educating patients to take ownership of their health and make educated decisions as health care consumers. As well as a lot of issues with costs associated with health care being opaque and difficult for people to understand and gain information about as they have decision making around their health. Yeah, I would say the biggest ones that come to mind is just inequities in the health care system and social determinants of health having huge impact on people's health care outcomes”
10	Participant #4: “Maybe due to our history, social, cultural, and political context, applying the same system as, for example Chile, without any adjustments might not be the most effective approach. So, the first step is to have a knowledge of the system, identify opportunities for improvement, and work towards strengthening and transforming it. Again, leadership is about generating transformation, change, but changes that truly benefit the majority of the population. It's not about making changes for the sake of it, as drastic implementation can sometimes do more harm than good. So, if one understands their healthcare system, its implementation, and its shortcomings, any transformation and change will indeed bring real benefits. I think this is what often happens - reforms and public policies might look good on paper, but when it comes to implementation, they may not deliver as expected. That's why we're constantly in a cycle of reform and policy modification. So, it's about responsible transformation based on knowledge”
11	Participant #articipant #26“there are multiple ways by which you can define leadership. You could at an individual level, right? I have written about moral courage. So, when you see something that's happening at the individual level with the patient that is not consistent with what our profession professes to be, having the courage to say no that's not what you should do. We should do something different, or we shouldn't cover up the mistake that we did. That's moral courage. That's leadership at the individual level. It's also leadership at the institutional level where decisions are made. And again, the ability to see what it is critical in healthcare. Care being the critical piece. And the ability to keep that excellence in patient care as the primary factor that determines everything else. Not based on how much more money can I get if I put the patient through really unnecessary procedures but it's about what is in the best interest of the patient. So, keeping that care central again requires leadership. So what I'm saying is I think leadership must be authentic. And courageous at the same time. Institutionality if you look at the system there are these health policy decisions that we have to make. Who gets what? How much do you cover? How much do not cover? And again, it has to be courageous and authentic. When we look at the big picture of our populations, it is not about an individual. What was valid for the population may not work for individual right? And in the US in particular, it's been all about individual care and I think we need to really emphasize population based and preventive care. I am a primary care doctor. Preventive care so that we don't get into all of these illnesses, but wellness, health is what we need to protect as a community. So, I see leadership at all those levels”
12	Participant #22: “Actually, I believe that everyone needs leadership competencies or capabilities. I think that the data will show that there are individuals who don't identify themselves as a leader. So, to me and some people will say, that they talk about this as “big L” or “little L” leader. I suppose, everybody needs be a little L leader, those are the things to influence others, to enact change, all those capabilities. And the bigger L leader is somebody who has formal roles. So, leader per se, the energy that has been expended to develop leadership curriculum, if you look at the literature, has been primarily for those who are in the leadership roles. Only recently, there have been more explorations in for students, residents and other healthcare professional students. Even in that sphere, so my university, for example, it's called “The president's leadership program” and it's, you know, for a select, you know, 25 people in the Province going to two universities. It's not just for health, it's for any learner at the undergraduate or graduate level, but they only take 25 each year. It's a resource intensive. it's great for those 25 people each year, but there's thousands and thousands of other students who also need to develop those capabilities. So, I think it's more important to think about it as leadership and, I guess, the flip side is followership, you know. You need to, as a follower, you need to be able to support the change and use all those same skills as if when you're the leader, earning that you still need to be able to speak up and to collaborate within a team. And I believe that the roles of leader and follower are fluid. Its fluid. We call that, everyday leadership, it's about the right person at the right time for the right problem. That individual can vary and isn't assigned that ability to lead that task or solve that problem, isn't tied to a specific person. It's about whoever is ready, capable and ultimately available to lead in that capacity. And I firmly believe that, hence, it's important for everybody to develop those capabilities”
13	Participant #17: “I kind of, after, you know, my 25 years of experience. I feel that not everybody will be or should be a leader. So I think that it's important. People could exhibit elements of leadership in different parts of their lives. That's great. But it may not be all of the students that will take on some very large leadership roles. So in my approach is I don't actually try to have 100% of the medical students behind all of our immigrant and refugee guidelines. And approaches I don't need 100% of students because I know that they will all not be working in this area. And they may develop other areas, which is fine. So I'm kind of in the 30% rule. So, you know, we often try to train up to 50 of our students. So I kind of believe the 30% rule and then from the 30% rule probably the 5% of those people, they actually become the inner circle of leaders in my way. So we work with a lot of student groups. And those student groups have to have student leaders. And I work with the student leaders. I don't work with the 50 students. But I work with the student leaders. They do all the training. They do all the work. And I just kind of mentor”
14	Participant #22: “The climate crisis, planetary justice, as well as equity access and participation in human rights, remain a global crisis, a public health crisis because of the impact. So whether that be food security, individuals experiencing homelessness, etcetera, in my world, and in pediatrics, the ACEs, or the adverse childhood events, all have an impact on health. And I think public health has a role to play for those”

**FIGURE 1:**
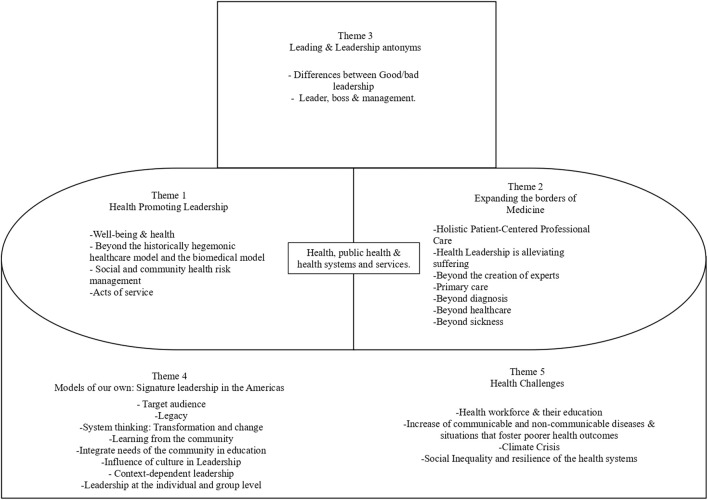
Framework for leadership in undergraduate medical education and training considering other professions. (Member states of the Pan American Health Organization. 2023).

### Health Promoting Leadership

“What I feel is more accessible is to make students health promoters. Teach them how to promote health. Here, for example, we have a group of public health professionals who focus on tobacco prevention, and they have a system called ABC that they easily introduce to undergraduate students” [excerpt: Participant #2].

This theme encapsulates the idea that the health workforce should play a role in promoting wellbeing and health, beginning with prevention and promotion strategies (quote 1). Additionally, it emphasizes the importance of developing leadership competencies in young learners by promoting health practices and teaching the community. Therefore, it is vital to conduct health risk assessments among individuals, families, and communities. When reflecting upon promoting health leadership, participants often highlighted the significance of self-awareness and personal development (quote 2). Furthermore, the ability to understand oneself and engage with others can be cultivated prior to pursuing undergraduate education.

### Expanding the Borders of Medicine

“Hospital systems were nonprofit, all of them, and they were in the business of helping people. When you had that kind of attitude. That permeates the system, you know, the doctors feel it, the nurses feel it, the janitors feel it. But when the change came and it kind of snuck up, you know, it didn't happen overnight, but it started happening. I think in the 80s and 90s. And you know now it’s just sort of overtaken the entire Healthcare System. You know the doctors, the nurses, they feel that the mission of the hospitals has changed and it’s not what they went to medical school to do”. [excerpt: Participant #10].

This theme reflects an expressed need to transform the current health systems and services, moving away from the traditional biomedical model. The shift is evident in the desire to move beyond disease-centric approaches and diagnoses, focusing instead on holistic health systems and services that incorporates primary healthcare principles. Emphasizing prevention and health promotion, this approach considers the social determinants of health and environmental determinants of health (quote 1).

### Leading and Leadership Antonyms

“You know authentic means that you live the life that you teach. I often think of Mahatma Gandhi’s quote. My life is my message. And I think that universities, teachers, and educators have a particular responsibility to demonstrate authentically the behaviors they espouse for their students.” [excerpt: Participant #23].

The conceptualization of leadership involves distinguishing it from other related but distinct concepts. Leadership should be differentiated from terms like leader, boss, and manager (quotes 3–5). A leader is a social position given to a person to exercise leadership. A boss may hold a position in an organization without necessarily possessing leadership competencies, while a manager may use resources to exercise leadership.

This theme addresses the essential characteristics necessary for effective leadership: utilizing diverse resources. It emphasizes that leadership is a process aimed at fostering collaboration and inspiring others to transform and address needs. Additionally, it explores the spectrum of leadership effectiveness (quote 6), distinguishing between good leadership (characterized by authenticity and positive influence towards constructive goals) and bad leadership (which negatively impacts others for malicious ends). Effective leadership requires individuals to possess appropriate qualifications and exercise their authority responsibly. Finally, leadership is viewed as a cultivated skill that can be enhanced through education, training, and competency development.

### Models of Our Own: Leadership Signature in the Americas With Latin America and the Caribbean

“For oneself, legacy is essential to the idea of leadership. Legacy is what one contributes to the development of an institution or organization. How one creates, adds, or contributes to an institution that exists before one arrives and will persist after one’s tenure in that institution ends. Legacy is what one leaves behind” [excerpt: Participant #30].

Legacy is another pivotal concept that emerged in the modeling of leadership in Latin America and the Caribbean. It emphasizes the importance of individuals understanding the legacy left within an organization that contributes to the mission, vision, strategies, and execution of plans (quote 7). Furthermore, system thinking for transformation and change was identified as incredibly important for the health systems and services within the Latin America and Caribbean context.

This theme underscores the unique characteristics of leadership in Latin America and the Caribbean, shaped by regional needs and experiences across historical, social, cultural, educational, and political contexts (quotes 6, 8–11). Additionally, it explores the notion that leadership can manifest in individuals from diverse academic backgrounds (quotes 12 and 13), emphasizing that leadership skills can be taught at both individual and collective levels (quote 11).

### *Challenges: Health, Public Health, and* Health Systems and Services

“The climate crisis, planetary justice, as well as equity access and participation in human rights, remain a global crisis, a public health crisis because of the impact. So, whether that be food security, individuals experiencing homelessness or the adverse childhood events, all have an impact on health. And I think public health has a role to play for those” [excerpt: Participant #22].

Identified challenges are closely related to health workforce education, which is characterized by a fragmented curriculum and insufficient utilization of problem-based learning. Furthermore, education primarily focuses on prescribing medication, creating a disconnect between UME and the actual job market (quote 8).

Social challenges also significantly impact health, contributing to the rise of various diseases, including oncological, neurodegenerative, and oral health issues, as well as crisis such as the opioid epidemic, gun violence, and climate change. The climate crisis, for instance, negatively affects human health through global pandemics, zoonoses diseases, and environmental factors like air pollution and rising temperatures, reduced access to clean water, increased exposure to harmful UV radiation from the sun, among others. Challenges within the health systems and services exacerbate inequities in population health, involving issues related to healthcare access, the sustainability of healthcare systems, and the intersection of politics with health policies.

The challenges are intricately linked to social determinants of health and environmental determinants of health, as evidenced by quotes 9 and 14. These determinants underscore disparities in healthcare access and health outcomes influenced by socioeconomic factors, as well as the interconnectedness between human health, the environment, and other living beings.

## Discussion

Interviews were conducted to explore the conceptualization of leadership in UME across Pan American Health Organization member states, with a focus on Latin America and the Caribbean. In contrast to previous studies by Keijser et al., Rajeh et al., Gordon et al., and the Canadian Medical Education Directives for Specialists which concentrated on single-country perspectives and primarily interviewed physicians, medical organizations, recognized leaders, and people working in hospitals or medical education [26–28, 40],our approach involved interviewing stakeholders across the Americas, who brought diverse expertise and academic backgrounds that could enrich the conceptualization of leadership for its use in teaching and training within UME, the health workforce, and other professions related to health and wellbeing (Supplementary Material 4).

### Conceptualization of Leadership and the Leader

Conceptualizations of leadership should begin by focusing on core themes related to education and training. In Canada, LEADS offers the following conceptualization: “Leadership is the collective capacity of an individual or group to influence people to work together to achieve a common constructive purpose: the health and wellness of the population we serve” [41]. We agree with LEADS in proposing that leadership should target the wellbeing and health of the population. These outcomes are fundamentally related to health systems and services and health promotion through collective risk management, which involves individuals, families and communities. The health systems and services is connected to patient care and individual risk management, while public health management involves social and environmental risk management. Both forms of risk management are intertwined with wellbeing, health, and health systems and services [Table 2: Bullet Points (BPs) 1].

**TABLE 2:** Leadership concepts in undergraduate medical education and training considering others. (Member states of the Pan American Health Organization.2023).

Bullet points (#)	Concepts
1	Leadership education and training should focus on the health systems and services that care for individuals, their families and communities. Health Promoting Leadership and expanding the borders of Medicine allows the health workforce (HWF) and the people to participate in individual, collective and public health risk management through the social and environmental health determinants in order to better well-being and improve health.
2	Leadership involves orchestrating resources and talents towards a shared vision, guiding transformative change, and fostering a culture of collaboration, empathy, and continuous learning to navigate challenges (theme about Challenges: Health, public health HHS) and achieve sustainable impact.
3	Leadership is a process of civic formation, especially in the case of the Health Workforce, like any professional, is foremost a person committed to societal well-being. This perspective underscores the importance of leadership fostering a sense of civic responsibility and community engagement.
4	Leadership involves the transformation of people through the actions of individuals with leadership competencies and positions. They leverage their own leadership competencies and positions to empower others to grow, not just follow. Their legacy lies in building a culture where leadership potential is nurtured throughout the society and organizations
5	Leadership is the practice of guiding and inspiring a community or organization through one's competencies and abilities. Unlike a boss, whose role is defined by organizational and social hierarchy, or a management, that is a component of leadership and relates to the allocation of resources (human, financial, time, etc.), a leader is a person and is recognized for their influence on others within their community or organization
6	Leadership is the ability to coordinate diverse groups towards a common goal while generating transformative change, making decisions that prioritize the well-being of individuals, and fostering critical thinking, along with empathy in problem-solving
7	Effective leadership encompasses the ability to inspire and empower others, facilitate open communication and active listening, and navigate complexities with humility, adaptability, and a deep understanding of the system to drive positive change and achieve collective goals
8	Leadership in the face of health challenges requires resilience, creativity, and a commitment to continuous improvement and lifelong learning, fostering a culture of adaptability, collaboration, and shared responsibility to address complex issues and create sustainable solutions for the greater good
9	Positive leadership embodies the ability to influence others positively, fostering collaboration, active listening, and continuous learning to achieve shared, beneficial goals while navigating and adapting to change with humility and adaptability
10	Negative leadership, in contrast, exerts detrimental influences on others, lacking empathy, critical thinking, and collaboration, often resulting in adverse consequences for individuals and the system at large
11	Effective leadership extends beyond authority, encompassing self-reflection to mitigate biases, establishing meaningful relationships, and promoting inclusivity and dialogue to understand and navigate complex systems for the betterment of all involved
12	Leadership extends beyond social authority, emphasizing self-awareness, ethical decision-making, and the ability to foster trust, collaboration, and innovation, achieving a higher performance and better societal impact
13	Leadership can be taught through competencies at both the individual and collective level
14	Leadership in health is characterized by the capacity to coordinate interdisciplinary teams, foster empathy and cultural competence, and promote holistic care, emphasizing prevention and well-being alongside interventions to address social and environmental determinants of health and achieve equitable outcomes

In contrast, LEADS primarily focuses on developing individual leadership competencies to improve the quality of healthcare service delivery [41]. Our conceptualization of leadership is centered on health-promoting leadership, which integrates individual, collective, and public health risk management. This integrated approach acknowledges pressing global issues such as the climate crisis and planetary justice (BP 2), recognizing the interconnectedness of human health with public health services and environmental determinants (e.g., the condition of water, land, air, flora, and fauna).

In Latin America and the Caribbean, Puertas et al. define leadership in Mexico as follows: “Leadership refers to the behaviors and actions that the leader takes to inspire, convince, or drive staff and the organization towards the achievement of the vision” [1]. However, we propose a multi-tiered approach to leadership development that begins with “leadership for people” training (BPs 1, 3 and 4), acknowledging that everyone is a person first. Subsequently, professions and occupations should incorporate leadership competencies relevant to their organizational roles. For instance, Jara et al. and Ibarra et al. have documented the integration of leadership education for people at the school level in Chile [42, 43]. Their research indicates that leadership education can foster identity formation and a sense of belonging among students, thereby enhancing competencies related to political participation. This focus on civic engagement is crucial, as highlighted by Pigg et al.’s study in the US, which examined the decline in voting rates among college-age individuals in presidential and congressional elections [44].

We concur with Pigg et al.’s assertion regarding the necessity of leadership education and training as a social process through which individuals develop community capacity to address challenges and mobilize resources for improved wellbeing and health [44]. Organizations cannot effectively address challenges without engaging the community in a leadership process that fosters meaningful and sustainable change.

Hartley et al. conceptualize healthcare leadership [45] by distinguishing between “leader development” and “leadership development.” The former focuses on developing individual human capital, assuming individual growth will lead to effective leadership. In contrast, leadership development is viewed as a social process involving relationships among individuals and organizations aimed at achieving common goals (BPs 3–6). In the Americas, frameworks like LEADS focus on broader leadership development across professions [41], while the Canadian Medical Education Directives for Specialists focuses on individual leader development. This is clearly described on its official website: “Canadian Medical Education Directives for Specialists is a physician competency framework that identifies and describes the abilities physicians require to effectively meet the healthcare needs” [40]. We disagree with the restrictive focus of the Canadian Medical Education Directives for Specialists framework, as we consider leadership to be a process that must cultivate competencies at both individual and collective levels to be capable of addressing the complex challenges in health, public health, and health systems and services.

The definition of leadership offered by Puertas et al. in Mexico, as the ability to inspire, convince, or drive individuals to achieve a vision [1], aligns with our conceptualization’s emphasis on inspiring, persuading, and guiding others toward a shared vision and goals related to wellbeing and health (BPs 2, 5, and 6). Our conceptualization builds upon this proposed definition and expands it by incorporating contextual nuances, challenges, and required capabilities, as proposed by Hartley et al. [45], which are crucial to operationalize political, economic, educational, and social frameworks as well. While the Mexican definition addresses organizational aspects within the social context, our conceptualization also emphasizes system thinking, exploring connections between the health systems and services and the broader context (BPs 7). Moreover, leadership should be framed within the context of challenges (BPs 2 and 8) [45], an aspect not consistently addressed in previous definitions [1, 41].

While Hartley et al. outline the capabilities necessary for effective leadership [36], definitions from frameworks like LEADS, Mexico, and Hartley generally assume a positive execution of leadership [1, 41, 45]. A comprehensive leadership conceptualization should encompass both effective and ineffective, along with positive and negative execution, grounded in values that guide ethical decision-making. These values include creativity, responsibility, empathy, active listening, assertive communication, humility, self-reflection, inclusivity, dialogue, trust, humility, adaptability, and resilience (BPs 2,3,6,7,9–12). Similarly, recognizing instances of negatively executed leadership is crucial (BPs 10), which is often characterized by the absence of these values and results in negative impacts on individuals. Therefore, leadership education and training should address both positive and negative leadership execution through formal, informal, and hidden education, utilizing both positive and negative role models for instruction.

### Matching the Conceptualization of Leadership With Competencies

Fitzpatrick et al. developed global competencies for the 12 Essential Public Health Functions [46], which are linked to challenges such as health inequities and strengthening prevention and promotion strategies, highlighting the importance of the social determinants of health and environmental determinants of health. Both Fitzpatrick et al. and our own conceptualization agree that these competencies should promote health equity among individuals and communities.

Our conceptualization of leadership is based on the premise that people equipped with leadership competencies represent the legacy and generational succession essential for the functional adaptation of societies to current and future challenges (BPs 4 and 13). We assert that leadership should have a positive impact on social determinants of health and environmental determinants of health, and that the health systems and services constitutes an intermediate determinant of social determinants of health (BP 14). Through addressing intermediate determinants, it becomes possible to impact structural determinants, such as the social, political, and economic contexts, as well as models of development, production, and consumption. Therefore, intersectoral and trans-sectoral collaboration is essential for addressing social determinants of health and environmental determinants of health.

Fitzpatrick et al. reinforce the importance of competencies related to ethics, collaboration, and lifelong learning [46]. We endorse their call by proposing that leadership development should ensure that individuals are equipped to manage resources and inspire others to work toward shared goals, guided by values, ethics, and professionalism. Additionally, it is vital to empower others to develop competencies that strengthen both individual and group performance (BPs 2, 4, 5, 6, 7, 8, 9, 10 12, and 13). Leadership development should not solely focus on professional improvement, but also include competencies related to civic responsibility and community engagement (BP 3).

This study followed the International Association for Health Professions Education for qualitative methods for health professions education [47, 48]. These guidelines recommend a participant range of 10–60 for qualitative studies, along with criteria for achieving data saturation. These conditions were met when we identified that no new information was emerging during interviews. Our sampling approach ensured representation from multiple institutional types, professional roles, and countries across the region and enabled a thick description. However, it must be acknowledged that most participants were from Mexico and Colombia, while potentially distinct perspectives from the Caribbean and Central American nations may not be captured. Additionally, most participants held positions in national academic institutions, with fewer voices from the health system, community sectors, or regional multilateral bodies. Despite these limitations, the insights gained offer transferability of findings to similar contexts across Pan American Health Organization member states by highlighting shared priorities, challenges, and opportunities for leadership education which can support decision-makers and researchers across the region. To build and strengthen this work, we recommend subsequent studies using quantitative, stratified approaches to capture the breadth of perspectives across the region.

### Recommendations

1. We invite the Pan American Health Organization member states to utilize the evidence derived from this project to inform policies regarding leadership education and training within the health workforce. The Andean Health Body–Hipólito Unanue Convention and the Ministries of Health from Venezuela, Colombia, Ecuador, Peru, Bolivia, and Chile have agreed to integrate these findings into education and training programs for both the health workforce and Ministries of Health personnel (Supplementary Material 6).
2. To achieve a lasting and significant impact on public health, it is imperative that medical education institutions and health policies prioritize and integrate the development of leadership competencies. This will not only better prepare the health workforce for current and future challenges, but also promote a more resilient, equitable, and efficient health systems and services.
3. The Pan American Health Organization member states must continue investing in the formation of leadership competencies in students. This is essential to ensure that graduates are not only clinically competent but are also capable of guiding and transforming health systems and services toward a healthier and more sustainable future.

### Priority Actions for Leadership Education and Training: The Pan American Health Organization

1. Academia: Within the curriculum, it is essential to work collaboratively with the Ministries of Health, the Ministry of Education, and relevant organizations involved in health workforce education to increase visibility of leadership conceptualization and its development through competencies within UME, the broader health workforce, and people. The intended outcome of this collaboration is that curricular guidelines explicitly include a definition of leadership, specific leadership competencies, and intended learning outcomes.
2. Governance Structures: Academia, people, and other stakeholders should participate in intersectoral governance spaces, such as committees dedicated to the formulation, implementation, evaluation, and revision of public policies. This active engagement is crucial for promoting leadership education and training within the health workforce.

### Conclusion

Our conceptualization of leadership for education and training in UME within the Pan American Health Organization member states has yielded several insights. First, it is structured around five themes ranked according to their importance for health, public health, and health systems and services, where the central themes have greater strength and intentionality in leadership education and training. This includes promoting leadership and expanding the borders of medicine to include health promotion and engage in systemic change that addresses the social determinants of health and environmental determinants of health. This approach ensures that leadership in UME within the Pan American Health Organization member states contributes meaningfully to improving population wellbeing and health across the Americas.

Second, our approach emphasizes the importance of distinguishing leadership from related concepts and underscores the ethical values essential for effective leadership. By focusing on attributes like resilience, empathy, and ethical decision-making, we aim to cultivate individuals capable of positively and effectively impacting health and health systems and services.

Third, our exploration highlights the critical need to integrate specific contextual challenges into leadership frameworks. Applying frameworks such as LEADS demonstrates that while universal leadership frameworks exist, their effectiveness largely depends on adaptation to national and cultural contexts.

## Data Availability

The datasets used and/or analyzed during the current study are available in the Supplementary Material, using the following link https://osf.io/mg5zq/?view_only=5f1714713e544a6bb108075d3bd99ea0 and from the corresponding author on reasonable request. The pre-print of this article can be read at: The Meaning of Leadership in Medical Education and Others in the Pan America Health Organization Member States: A Stakeholder Analysis and Interviews | Research Square [49].
