# Supplementary material for: The Meaning of Leadership in Medical Education in the Pan American Health Organization Member States: A Stakeholder Analysis and Interviews
Source: Int J Public Health. 2026 Feb 26;71:1608502. doi: 10.3389/ijph.2026.1608502 (PMC12979235; doi:10.3389/ijph.2026.1608502)
Supplement: Supplementary file 1 [file Supplementaryfile6.docx]

| **Andean Human Resources Committee Meeting**  **June 12, 2024** |
| --- |

| Goal | To propose a regional study on developing leadership competencies in Andean countries. |
| --- | --- |
| Date | June 12th, 2024 |
| Time | 9:00 hr Colombia, Ecuador, Perú  10:00 hr Bolivia, Chile, Venezuela |
| Location | Online meeting on Zoom, organized by ORAS-CONHU |

**List of attendees:**

| Name | **Charges** | **Country/Organization** |
| --- | --- | --- |
| Dra. Silvia Cuencas | Human Resources Unit – MSD Bolivia | Bolivia |
| Dra. Raquel Child | Head of the Office of Cooperation and International Affairs (OCAI) – MINSAL Chile | Chile |
| Dra. Olga Correa | Head of the Department of Planning and Control of Human Resources Management in Health – MINSAL Chile | Chile |
| Dr. Fredy Carmelo Tovar | Directorate of Human Talent Development in Health - MSPS Colombia | Colombia |
| Dr. Andrés Molina | Specialist in the formulation of standards and profiles of human talent in health – MSP Ecuador | Ecuador |
| Dr. Dr. Carlos Espinoza Barreto | General Directorate of Health Personnel – MINSA Perú | Perú |
| Lic. Militza Aguilar | Area of ​​Classification and Remuneration of Positions, Directorate General of Research and Education – MPSS Venezuela | Venezuela |
| Dr. Pablo Rodríguez | Fac. Health, Medicine and Life Sciences - Maastricht University | Maastricht University |
| Dra. Katarzyna Czabanowska | Fac. Health, Medicine and Life Sciences - Maastricht University | Maastricht University |
| Dra. María del Carmen Calle Dávila | Executive Secretary | ORAS-CONHU |
| Dra. Marisella Mallqui Osorio | Deputy Secretary | ORAS-CONHU |
| Mg. Yadira Salas | Coordinator of the Andean Committee on Human Resources in Health | ORAS – CONHU |
| Stephany Tafur Contreras | Public Health and Global Health Professional | ORAS – CONHU |

**SCHEDULED**

The agenda for the meeting was as follows:

- Opening remarks by Dr. María del Carmen Calle Dávila, Executive Secretary of ORAS-CONHU.
- Opening remarks by Dr. Katarzyna Czabanowska (K.M.), International Health School CAPHRI, Care & Public Health Research Institute, Faculty of Health, Medicine and Life Sciences, Maastricht University, The Netherlands.
- Presentation of the regional study proposal by Dr. Pablo Rodríguez, Maastricht University, The Netherlands.
- Country interventions and dialogue between the presenters and the members of the Andean Human Resources Committee.
- Closing remarks by Dr. María del Carmen Calle Dávila, Executive Secretary of ORAS-CONHU.

**AGENDA DEVELOPMENT**

- - **Opening remarks by Dr. María del Carmen Calle Dávila, Executive Secretary of ORAS-CONHU.**
  - **Highlighting** the need to develop strategies that strengthen governance and stewardship as a primary action line of the Andean human resources policy, promoting a consensus on policies and strategies that balance the training of health personnel with professional life courses.
  - **Emphasizing** that the Andean policy is a provision of human resources that can respond to a changing reality in epidemiological, environmental, demographic, and socioeconomic terms.
  - **Recalling** that, in order to comply with the mandates of the Andean Ministers of Health and the objectives of the Andean HRH policy, a letter of understanding has been signed between Maastricht University, through the International Health School CAPHRI, Care and Public Health Research in Faculty Health, Medicine and Life Sciences, and ORAS-CONHU.
  - **Both institutions** share the vision of providing evidence-based perspectives and recommendations to shape a sustainable, sized, and continuously trained workforce, committed to the quality and relevance of health services.
  - **Collaboratively**, both institutions plan to carry out the leadership project for human resources in health, in order to make the health-education binomial their responsibility to accelerate a more effective response and preparedness with competencies in institutional articulations, facilitation of participatory processes, and committed to the effective exercise of the right to health of the communities they serve.
  - **Opening remarks by Dr. Katarzyna Czabanowska (K.M.), International Health School CAPHRI, Care & Public Health Research Institute, Faculty of Health, Medicine and Life Sciences, Maastricht University, The Netherlands.**
    - **Highlighting** significant milestones achieved through institutional partnerships in the European region and other parts of the world that could be adapted to specific contexts, with a particular interest in public health leadership and workforce development.
    - **Introducing herself** as a WHO expert in the area of public health leadership, supporting the development of public health leadership courses for health professionals and the workforce, as well as the development and professionalization of health workforces in support of UHC.
    - **Presenting** WHO documents developed in partnership with stakeholders such as National Public Health Institutes, associations, and schools to support the workforce:
    - The Roadmap for the Professionalization of the Health Workforce in the European Region.
    - The Competency Framework for the Public Health Workforce outlining the core competencies that public health professionals should possess.
    - The Roadmap for Emerging Workforces.
    - Competencies to Support Education and Leadership in Public Health.
  -
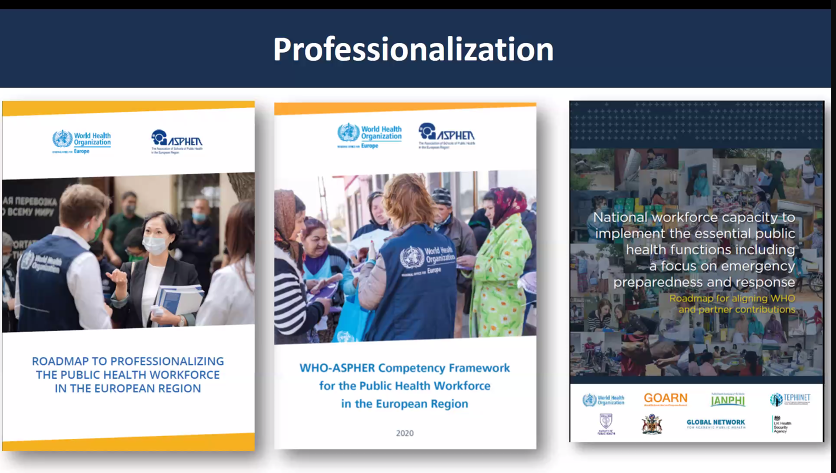

  - **Presentation of the regional study proposal by Dr. Pablo Rodríguez, Maastricht University, The Netherlands.**
- The proposal addresses leadership in health human resources with an interprofessional, transprofessional, and citizen-centered approach.
- Various specific frameworks regarding leadership competencies and health leadership, developed in the United Kingdom for the European context and adaptable to other contexts, were presented:
  - The project, in 2023, began developing the following::
  - The results of the first phase of the project were summarized as follows:
  - Health challenges: healthcare services, public health operations, one health, and education.
  - Leadership education: health workforce, health governance, and civic education.
  - Education: competencies to address health challenges.
  - The conclusions of the first phase of the project were summarized as follows:
  - There are competency frameworks for leadership in the health workforce and governance.
  - Conceptualization of leadership in Latin America.
  - Competencies for health leadership in Latin America.
  - Applying knowledge: teaching health leadership in Andean countries.
    - The following actions for the project are proposed:
      - Initiate knowledge translation activities.
      - Determine the most demanded leadership competencies in Andean countries at two levels:
    - Health ministries responsible for governance.
    - Health workforce providing healthcare services.
    - The methodology to be used would be to conduct a survey to study leadership competencies at the two mentioned levels.
    - The purpose is to avoid research waste (the gap between knowing and doing) once leadership competencies have been established.
    - Develop material in Spanish for teaching leadership competencies.

**AGREEMENTS**

Following the presentation of the proposal, a round of consultations with countries and relevant entities was conducted to gather their feedback on the proposal:

| PAÍS | **POSICIÓN** |
| --- | --- |
| **Bolivia** | - The proposal was approved. - They have a Community-Intercultural Family Health Policy in place, which aims to address the social determinants of health. - They recognize the need for a multidisciplinary team to address the social determinants of health with health leadership. - The policy has been disseminated to health professionals; it is expected to be disseminated to other health-related professionals. - They consider it important to add certification value to the proposed training. |
| **Chile** | - The proposed initiative was approved. - Governance is a top priority and challenge for them. |
| **Colombia** | - The proposed initiative was approved. - They consider it necessary to address public health capacities beyond operational aspects, focusing on new ways of conceiving and conducting health policies for the common good and good governance. - From a governance perspective, they consider it important to regulate dynamics related to health education, the labor market, and the provision of health services. |
| **Ecuador** | - The proposal has been approved. - They are in the process of developing a National Human Resources Policy. - They consider it important to incorporate the health workforce leadership framework into national policies related to human resources. |
| **Perú** | - The proposal has been approved. - From a governance perspective, they consider the proposal to be aligned with national policies related to health human resources. |
| **Venezuela** | - The proposed initiative has been approved. |
| **ORAS-CONHU** | - The proposed initiative has been approved. - It is agreed to follow and execute the operational roadmap presented for the second phase of the proposal. - A follow-up meeting has been scheduled to discuss the methodological tools to be addressed in the second phase. - The importance of a collaborative learning approach is emphasized. |
