# Supplementary material for: The Meaning of Leadership in Medical Education in the Pan American Health Organization Member States: A Stakeholder Analysis and Interviews
Source: Int J Public Health. 2026 Feb 26;71:1608502. doi: 10.3389/ijph.2026.1608502 (PMC12979235; doi:10.3389/ijph.2026.1608502)
Supplement: Supplementary file 2 [file Supplementaryfile1.docx]

**Supplementary material 1. Operational definitions**

| **#** | **Definition in English** | **References** |
| --- | --- | --- |
| 1 | **Collective Risk Management:** The management of health risks in collective interventions and communities through the implementation of territorial health plans, execution of collective intervention plans, and other tools. | Gobierno de Colombia. Ministerio de Salud y de la Proteccion Social. Gestión integral de riesgo en salud. 2018  [Gestión integral de riesgo en salud (minsalud.gov.co)](https://www.minsalud.gov.co/proteccionsocial/Paginas/gestion-integral-de-riesgo-en-salud.aspx) Accessed 21 August 2024 |
| 2 | **Health**: The ability of individuals or communities to adapt and self-manage the physical, mental, or social challenges they encounter in life. | Gobierno de Colombia. Ministerio de Salud y de la Proteccion Social. 2020. [“Debemos ser capaces de adaptarnos juntos”: Alejandro Jadad (minsalud.gov.co)](https://www.minsalud.gov.co/Paginas/Debemos-ser-capaces-de-adaptarnos-juntos-Alejandro-Jadad.aspx?fbclid=IwZXh0bgNhZW0CMTAAAR2vFdugFdMGu9XqIcC43f_GHyRhyFBGQXS7YZBFpZHY_CB5rzTHlLlNSDY_aem_jaTzKGNgChpYMhXzHaMJWA) Accessed 21 August 2024 |
| 3 | **Health systems and services**: The primary objective of the Department of Health Systems and Services is to strengthen health systems based on Primary Health Care, support the transformation of national health systems to improve equity and resilience, strengthen Primary Health Care (PHC), and address the relevant issues related to human resources as health systems move towards the achievement of Universal Health. Its work encompasses the following areas: integrated services delivery, strengthening of health systems governance and stewardship; developing efficient and equitable financial mechanisms to ensure adequate public funding of the health system; human resources for health, healthy life course; the Virtual Campus of Public Health, and Latin American Center of Perinatology, Women and Reproductive Health (CLAP). | Pan American Health Organization. Health Systems and Services. 2024. <https://www.paho.org/en/health-systems-and-services> Accessed 21 August 2024. |
| 4 | **Health workforce:**   1. Generalist medical practitioners. 2. Specialist medical practitioners. 3. Nursing professionals. 4. Midwifery professionals 5. Dentists 6. Pharmacists 7. Physiotherapists 8. Social work and counselling professionals 9. Life science professionals | World Health Organization. Classifying health workers: Mapping occupations to the international standard classification. <https://cdn.who.int/media/docs/default-source/health-workforce/dek/classifying-health-workers.pdf?sfvrsn=7b7a472d_3&download=true> Accessed 21 August 2024. |
| 5 | **Individual Risk Management:** The individual management of risk through the identification, evaluation, measurement, treatment, tracking, and monitoring of risks that may compromise the health of the population, with the aim of delivering health services and technologies comprehensively to promote health, prevent, treat, rehabilitate, palliate, or cure disease. | Gobierno de Colombia. Ministerio de Salud y de la Proteccion Social. Gestión integral de riesgo en salud. 2018  [Gestión integral de riesgo en salud (minsalud.gov.co)](https://www.minsalud.gov.co/proteccionsocial/Paginas/gestion-integral-de-riesgo-en-salud.aspx) Accessed 21 August 2024 |
| 6 | **Interprofessional education:** involves students of two or more professions learning together, especially about each other’s roles | Frenk J, Chen L, Bhutta ZA, et al. Health professionals for a new century: transforming education to strengthen health systems in an interdependent world. Lancet. 2010; [https://doi.org/10.1016/S0140‐6736(10)61854‐5](https://doi.org/10.1016/S0140%E2%80%906736(10)61854%E2%80%905) |
| 7 | **Latin America and the Caribbean:** The geographic area of Mexico, Central America, continent of South America and the islands of the Caribbean where Spanish or other Romance language is spoken. The area that lies between continental North and South America and comprises the Caribbean Sea, the West Indies, and the adjacent mainland regions of southern Mexico, Central America, Colombia, and Venezuela. | National Library of Medicine <https://meshb.nlm.nih.gov/record/ui?ui=D007843>  <https://meshb.nlm.nih.gov/record/ui?ui=D017691> Accessed 21 August 2024 |
| 8 | **People:** human beings; men, women and children | Oxford. People.2024. [people noun - Definition, pictures, pronunciation and usage notes \| Oxford Advanced Learner's Dictionary at OxfordLearnersDictionaries.com](https://www.oxfordlearnersdictionaries.com/us/definition/english/people_1?q=people) Accessed 21 August 2024. |
| 9 | **Public health leadership competency framework model:** The frameworks contain eight domains and 52 competencies. The domains are Systems thinking (D1), Political leadership (D2), Collaborative leadership: building and leading interdisciplinary teams (D3), Leadership and communication (D4), Leading change (D5), Emotional intelligence and leadership in team-based organizations (D6), Leadership, organizational learning and development (D7), and Ethics and professionalism (D8). | Czabanowska K, Smith T, Könings KD, Sumskas L, Otok R, Bjegovic-Mikanovic V, et al. In Search for a Public Health Leadership Competency Framework to Support Leadership Curriculum-A Consensus Study. Eur J Public Health. 2014; <https://doi.org/10.1093/eurpub/ckt158> |
| 10 | **Public Health Risk Management:** The management of public health within a territory by addressing social and environmental risks. | Gobierno de Colombia. Ministerio de Salud y de la Proteccion Social. Gestión integral de riesgo en salud. 2018  [Gestión integral de riesgo en salud (minsalud.gov.co)](https://www.minsalud.gov.co/proteccionsocial/Paginas/gestion-integral-de-riesgo-en-salud.aspx) Accessed 21 August 2024 |
| 11 | **The Pan American Health Organization member states:**   1. Antigua and Barbuda 2. Argentina 3. Bahamas 4. Barbados 5. Belize 6. Bolivia 7. Brazil 8. Canada 9. Chile 10. Colombia 11. Costa Rica 12. Cuba 13. Dominica 14. Dominican Republic 15. Ecuador 16. El Salvador 17. Grenada 18. Guatemala 19. Guyana 20. Haiti 21. Honduras 22. Jamaica 23. Mexico 24. Nicaragua 25. Panama 26. Paraguay 27. Peru 28. Saint Lucia 29. St. Vincent and the Grenadines 30. St. Kitts and Nevis 31. Suriname 32. Trinidad and Tobago 33. United States of America 34. Uruguay 35. Venezuela  \|  \| \| --- \| | Pan American Health Organization. Member States of the Pan American Health Organization. 2024. [Member States of the Pan American Health Organization - PAHO/WHO \| Pan American Health Organization](https://www.paho.org/en/planning-finance-and-accountability/member-states-pan-american-health-organization) Accessed 21 August 2024. |
| 12 | **The Americas**: The general name for NORTH AMERICA; CENTRAL AMERICA; and SOUTH AMERICA unspecified or combined. | National Library of Medicine. Americas. 2024  <https://www.ncbi.nlm.nih.gov/mesh/?term=the+americas> Accessed 21 August 2024. |
| 13 | **Transprofessional education:**  That includes non-professional health workers might be of even greater importance for health-system performance, especially the teamwork of professionals with basic and ancillary health workers, administrators and managers, policy makers, and leaders of the local community | Frenk J, Chen L, Bhutta ZA, et al. Health professionals for a new century: transforming education to strengthen health systems in an interdependent world. Lancet. 2010; [https://doi.org/10.1016/S0140‐6736(10)61854‐5](https://doi.org/10.1016/S0140%E2%80%906736(10)61854%E2%80%905) |
| 14 | **Undergraduate medical education:** the period beginning when a student enters medical school and ends with the final examination for basic medical qualification. This period of education comprises a pre-clinical and a clinical period. | Accreditation Council of the Eurasian Centre for Accreditation and Quality Assurance in Higher Education and Health Care. Glossary of medical education terms  [Glossary of Medical Education Terms (engl).pdf (ecaqa.org)](https://www.ecaqa.org/doxs/Glossary%20of%20Medical%20Education%20Terms%20(engl).pdf) Accessed 21 August 2024. |
| 15 | **Well-being:**  This concept has four capitals   1. Workforce well-being increases performance, decreases staff turnover, and has positive outcomes for employees, employers and the overall economy 2. Human well-being: People’s health and their subjective well-being are closely linked; both are drivers of economic prosperity. 3. Planetary well-being: The environment is key to human well-being and economic prosperity 4. Social well-being: Trust, participation and social cohesion make significant contributions to health and well-being, including health equity, as well as being vital to prosperous and resilient societies. | Health in the well-being economy. Background paper: working together to achieve healthy, fairer, prosperous societies across the WHO European Region. Copenhagen: WHO Regional Office for Europe. 2023. Licence: CC BY-NC-SA 3.0 IGO. Available from: [Health in the well-being economy: background paper: working together to achieve healthy, fairer, prosperous societies across the WHO European Region](https://www.who.int/europe/publications/i/item/WHO-EURO-2023-7144-46910-68439) Accessed on January 19^th^ 2025 |
