# Supplementary material for: The Meaning of Leadership in Medical Education in the Pan American Health Organization Member States: A Stakeholder Analysis and Interviews
Source: Int J Public Health. 2026 Feb 26;71:1608502. doi: 10.3389/ijph.2026.1608502 (PMC12979235; doi:10.3389/ijph.2026.1608502)
Supplement: Supplementary file 3 [file Supplementaryfile5.docx]

**Supplementary material 5 . Coding: Themes, subthemes, codes and participants´ quotes**

Theme 1.0 Health Promoting Leadership

| **Organization of the Coding** | **Interpretation and Important Quotes** |
| --- | --- |
| 1. Theme: Health Promoting Leadership | |
| Health Promoting Leadership | Students in health-related professions should be able to promote well-being and health. It starts with prevention and promotion.  Participant #2: “*At least what I feel is more accessible is to make students health promoters. Teach them how to promote health. Here, for example, we have a group of public health professionals who focus on tobacco prevention, and they have a system called ABC that they easily introduce to undergraduate students. Most of our students from all disciplines have the opportunity to do rotations in primary care, and a significant part of what they are taught and experience there is related to prevention. In our case, for example, issues like tobacco prevention and oral hygiene are basic topics that we teach and promote extensively in undergraduate education. However, students encounter a completely different reality when they enter the workforce. In practice, there is often more emphasis on treating diseases than preventing them. We are constantly trying to promote a different approach as healthcare professionals, but the reality of the job market often clashes with that*”. |
| Codes: Health promoting leadership | |
| Well-being and health | According to Psychology, “well-being is the experience of health, happiness, and prosperity. It includes having good mental health, high life satisfaction, a sense of meaning or purpose, and the ability to manage stress. More generally, well-being is just feeling well”. |
| Beyond the historically hegemonic healthcare model and the biomedical model | It emphasizes the need to move beyond the traditional healthcare model, historically hegemonic biomedical model to consider other treatment options and conceptualizations of health. It recognizes the need to appraise different cultures and their world views into health |
| Social and community health risk management | Putting the needs of individuals, families and community first in well-being and health through individual, collective, and public health risk management. This involves collaboration among the HWF and other sectors to manage health risks from prevention to palliative care, aiming to prevent diseases and injuries or detect them early to mitigate their impact, ultimately improving population health, user experience, and cost efficiency.  Participant #2: “It's a teaching and learning methodology that involves meeting the needs of a community partner, as it's called, and addressing a problem that originates from the community itself. It's not about the university inventing the problem or tailoring it to fit their needs. Instead, the community identifies the issue that we will support, and students learn by doing something that benefits the community”. |
| Acts of service | Understanding the health care profession’s work as caring for others and their actions as being in the service of people |

Theme 2.0 Expanding the borders of medicine.

| **Organization of the Coding** | **Interpretation and Important Quotes** |
| --- | --- |
| 1. Theme: Expanding the borders of medicine | |
| Expanding the borders of medicine | The need to redirect the current health systems based on the biomedical model. |
| Codes: Expanding the borders of Medicine | |
| Beyond Diagnosis | An approach to patient care that prioritizes well-being and health over symptom reduction and reducing the patient to a disease.  Participant #1: “*"you have to ask the patient how he feels, then you have to ask him where he lives and if there are pets in his house and how many people live with him etc.… like all these aspects that people say, but what does it have to do with pain. And of course, this is a tenacious shock because when you go to the doctor you are not asked that. They ask you when and where your head hurts, since when does it hurt, can you move your eyes and nose, ah ready, take this. So, I think that I see how these kids suddenly ask the question: What happens if I suggest that they sleep an extra hour a day or what happens when I tell the lady, hey, how difficult it must be to have the responsibility of two children. and also have a headache as severe as the one you are telling me about*”. |
| Beyond Healthcare | The idea patientcare should go beyond traditional healthcare: Preventing and promoting well-being and health.  Participant #10: “Sure, so it used to be. Hospital systems were nonprofit all of them and that they were in the business of helping people. When you had that kind of attitude. That permeates the system, you know, the doctors feel it, the nurses feel it, the janitors feel it. You are there to help people get better. And if they can't help people get better, they will help them have a good end of life. You know, as best as possible. But when the change came and it kind of snuck up, you know, it didn't happen in overnight, but it started happening. started happening. I think in the 80s and 90s. And you know now it's just sort of overtaken the entire Healthcare System, nearly the entire Healthcare System. You know the doctors the nurses, the staff, they feel that they feel that the mission of the hospitals have changed and it's not what they went to medical school to do. It’s not what they were trained to do” |
| Beyond sickness | Refers to treating patients’ sickness as more than disease. |
| Holistic Patient-Centered Professional Care | Contrary to a model that prioritizes and overvalues medical subspecialisation, this model takes a holistic view on patient care. |
| Health Leadership is alleviating suffering | The idea that leadership within the healthcare system aims to alleviate suffering. |
| Beyond the creation of experts | A need of having professionals that can attend to primary care needs instead of specialized care |
| Primary Care | A care model based on prevention and promotion, considering social and environmental determinants of health. |

Theme 3.0 Leading & Leadership antonyms: themes, subthemes and codes

| **Organization of the Coding** | **Interpretation and Important Quotes** |
| --- | --- |
| 1. Theme: Leading & Leadership antonyms | |
| Leading & Leadership antonyms | Characteristics of a person that allow them to lead and manage various resources (human, economic, technological, temporal) towards a goal. There are concepts that are used interchangeably from leadership, even though these concepts are different from leadership. The type of leadership to be executed is strictly related to the context and its needs.  Participant #4: “*Maybe due to our history, social, cultural, and political context, applying the same system as, for example Chile, without any adjustments might not be the most effective approach. So, the first step is to have a knowledge of the system, identify opportunities for improvement, and work towards strengthening and transforming it again*”. |
| 3.1 Subtheme: Good/bad leadership | |
| Good/bad leadership | Good: leadership characterized by positively influencing others and achieving positive goals  Bad: leadership characterized by negatively influencing others resulting in negative consequences in others and the goals.  Participant #23: *“I think this is a matter of what I would call authenticity. Pablo, you know authentic means that you live the life that you teach. I often think of Mahatma Gandhi's quote. My life is my message. My life is my message. And I think that universities and teachers and educators have a particular responsibility to demonstrate authentically the behaviors of their spousing for their students. That means being a great doctor being. Being a great doctor means, I think, being socially responsible as well as caring for individuals and your patients. I think that there's really no room, in my view, for hypocrisy and that regard you're describing hypocrisy among political leaders. That you know profit off of war and battles and so on. Now, you know having said that people are people and people are complex, right and you'll always have some degree of misbehavior or some”.* |
| Codes: Good/bad leadership | |
| Leadership seen as positive/negative | The idea that people can have a positive or negative influence on others while they are executing leadership roles. |
| Laissez-faire | Leadership with negative influences on others and/or harmful expected or unexpected consequences a possible consequence of not including leadership competencies explicitly in the formal curriculum. |
| Leadership is about responding to needs | Understanding that leadership aims to give solution to real people’s problems and answer to their needs.Answering the needs of others may result in either good or bad leadership depending on the effects had. |
| Leadership is a cultivated habit | Understanding that leadership competencies can be learned and improved over time with the correct supervision and mentoring. This means that educators influence the development of leadership competencies in students to develop good or bad leadership through formal and informal practices. |
| 3.2 Subtheme: Differentiating from Leader | |
| Differentiating from Leader | The leader is understood as a social position given to a person within a community to exercise leadership competencies.  Participant #27: “in my mind leader is a person. Leadership is about the qualities and skills of the person that allows for change to happen. It's really about making change happen if they're in a positive direction”. |
| Code: Differentiating from Leader | |
| Leaders are not just authority figures | The belief that leadership is more than a social position or status symbol of authority |
| 3.3 Subtheme: Differentiating from Boss | |
| Differentiating from Boss | A boss is interpreted as a position within an organization and is not related to having competencies or abilities.  Participant #07: *“For example, we could say it's one thing to have a boss who bosses you around and says, “you have to do this and this and this and this and this because I said so and that's the way it is”, right? And a leader who is usually someone who inspires you, who sometimes doesn't necessarily have to be saying “do this because I say so” but people follow just because he has the condition and the skills to do it, so that's what's left. Leadership terms could be misunderstood in that aspect, to be leaders by forcing people to follow instead of generating a positive influence in the group so that the group is convinced of your message of the need to make a change”.* |
| Codes: Differentiating from Boss | |
| Power differences | Understanding that any system can have power differences within people that affect leadership |
| Role qualifications | Set of qualifications required by a person to perform in a leadership position |
| 3.4 Subtheme: Differentiating from Management | |
| Differentiating from Management | Resource management is one component and competency of leadership.  Participant #30: *“Well, for me, they are two sides of the same coin. They are usually contrasted, but I actively believe that they are not opposed. Leadership without management becomes ineffective leadership that never translates into practice, and management without leadership is like moving forward without advancing, without a clear direction. So, leadership creates a destination, and management builds a path to that destination. You cannot have one without the other. While we typically contrast them, I believe that is a conceptual mistake. For me, they are two indispensable components. There are managers who are not leaders, that's true. But they are part of a team where there is a shared vision articulated by him or the leaders. So, it's not so much that each individual has to be simultaneously a leader and a good manager, but that an organization, an institution, a community has to have both components. They reinforce each other”.* |
| Code: Differentiating from Management | |
| Resource management | Being able to effectively coordinate all sorts of resources towards a goal (human, financial, time, etc.) |

Theme 4.0 Models of our own: Leadership signature in the Americas using a Latin America and the Caribbean approach.

| **Organization of the Coding** | **Interpretation and Important Quotes** |
| --- | --- |
| 4. Theme: Models of our own: Leadership signature in the Americas with LAC focus | |
| Models of our own: Leadership signature in the Americas with LAC focus. | The need to create in the PAHO MS, including LAC models bases on our own needs and experiences  Participant #4: *“Maybe due to our history, social, cultural, and political context, applying the same system as, for example Chile, without any adjustments might not be the most effective approach. So, the first step is to have a knowledge of the system, identify opportunities for improvement, and work towards strengthening and transforming it. Again, leadership is about generating transformation, change, but changes that truly benefit the majority of the population. It's not about making changes for the sake of it, as drastic implementation can sometimes do more harm than good. So, if one understands their healthcare system, its implementation, and its shortcomings, any transformation and change will indeed bring real benefits. I think this is what often happens – reforms and public policies might look good on paper, but when it comes to implementation, they may not deliver as expected. That's why we're constantly in a cycle of reform and policy modification. So, it's about responsible transformation based on knowledge”.* |
| Codes Models of our own: Models of our own: Leadership signature in the Americas with LAC focus | |
| Who should be taught Leadership? | Refers to the need of developing leadership competencies in a broader population not just the physicians or certain professions. It explores the question of is leadership innate in a person? Or can it be developed? The main conclusion among participant is that even though one may be born with some leadership attributes or not, leadership competencies can and should be developed in the Health Workforce. Furthermore, it relates to leadership at the individual and group levels.  Participant #27: *“there are multiple ways by which you can define leadership. You could at an individual level, right? I have written about moral courage. So, when you see something that's happening at the individual level with the patient that is not consistent with what our profession professes to be, having the courage to say no that's not what you should do. We should do something different, or we shouldn't cover up the mistake that we did. That's moral courage. That's leadership at the individual level. It's also leadership at the institutional level where decisions are made. And again, the ability to see what it is critical in healthcare. Care being the critical piece. And the ability to keep that excellence in patient care as the primary factor that determines everything else. Not based on how much more money can I get if I put the patient through really unnecessary procedures but it's about what is in the best interest of the patient. So, keeping that care central again requires leadership. So what I'm saying is I think leadership must be authentic. And courageous at the same time. Institutionality if you look at the system there are these health policy decisions that we have to make. Who gets what? How much do you cover? How much do not cover? And again, it has to be courageous and authentic. When we look at the big picture of our populations, it is not about an individual. What was valid for the population may not work for individual right? And in the US in particular, it's been all about individual care and I think we need to really emphasize population based and preventive care. I am a primary care doctor. Preventive care so that we don't get into all of these illnesses, but wellness, health is what we need to protect as a community. So, I see leadership at all those levels*”.  Participant #22: *“Actually, I believe that everyone needs leadership competencies or capabilities. I think that the data will show that there are individuals who don't identify themselves as a leader. So, to me and some people will say, that they talk about this as `big L´ or `little L´ leader. I suppose, everybody needs be a little L leader, those are the things to influence others, to enact change, all those capabilities. And the bigger L leader is somebody who has formal roles. So, leader per se, the energy that has been expended to develop leadership curriculum, if you look at the literature, has been primarily for those who are in the leadership roles. Only recently, there have been more explorations in for students, residents and other healthcare professional students. Even in that sphere, so my university, for example, it’s called “The president's leadership program” and it's, you know, for a select, you know, 25 people in the Province going to two universities. It’s not just for health, it’s for any learner at the undergraduate or graduate level, but they only take 25 each year. It’s a resource intensive… it's great for those 25 people each year, but there's thousands and thousands of other students who also need to develop those capabilities. So, I think it's more important to think about it as leadership and, I guess, the flip side is followership, you know. You need to, as a follower, you need to be able to support the change and use all those same skills as if when you're the leader, earning that you still need to be able to speak up and to collaborate within a team. And I believe that the roles of leader and follower are fluid. Its fluid. We call that, everyday leadership, it’s about the right person at the right time for the right problem. That individual can vary and isn't assigned that ability to lead that task or solve that problem, isn't tied to a specific person. It's about whoever is ready, capable and ultimately available to lead in that capacity. And I firmly believe that, hence, it's important for everybody to develop those capabilities*”  Participant #17: “*I kind of, after, you know, my 25 years of experience. I feel that not everybody will be or should be a leader. So I think that it's important. People could exhibit elements of leadership in different parts of their lives. That's great. But it may not be all of the students that will take on some very large leadership roles. So in my approach is I don't actually try to have 100% of the medical students behind all of our immigrant and refugee guidelines. And approaches I don't need 100% of students because I know that they will all not be working in this area. And they may develop other areas, which is fine. So I'm kind of in the 30% rule. So, you know, we often try to train up to 50 of our students. So I kind of believe the 30% rule and then from the 30% rule probably the 5% of those people, they actually become the inner circle of leaders in my way. So we work with a lot of student groups. And those student groups have to have student leaders. And I work with the student leaders. I don't work with the 50 students. But I work with the student leaders. They do all the training. They do all the work. And I just kind of mentor*”. |
| Legacy | Refers to what is contributed or developed within an organization that persists over time, even after personally leaving the organization. Without a clear understanding of the legacy, it's difficult to articulate the mission/vision, strategy, and execution plans within an organization.  Participant #30: “*And this requires the leader to have a clear sense of legacy. For oneself, legacy is essential to the idea of leadership. Legacy is what one contributes to the development of an institution or organization. How one creates, adds, or contributes to an institution that exists before one arrives and will persist after one's tenure in that institution ends. Legacy is what one leaves behind, and that is the key concept of leadership. Without a clear legacy concept, it is very difficult to activate the vision, strategy, and execution plan that I mentioned before*”. |
| System thinking: Transformation and change | Assuming that leadership is oriented towards transforming and changing the status quo for the better. This can only be achieved after understanding the sociopolitical, economic, educational, and organizational contexts influences in the health system |
| Learning from the community | Refers to the possibility to learn from a community. |
| Integrate needs of the community in education | The need to consider community needs as part of healthcare |
| Influence of culture in Leadership | The creation of psychologically safe spaces for people from different communities |
| Leadership is context-dependent | Assuming leadership roles depends largely on the needs of a situation or context, meaning leadership positions can fluctuate within a group. |
| Leadership at the individual and group level | The idea that leadership competencies are executed both the individual and collective level.  Participant #26:*“there are multiple ways by which you can define leadership. You could at an individual level, right? I have written about moral courage. So, when you see something that's happening at the individual level with the patient that is not consistent with what our profession professes to be, having the courage to say no that's not what you should do. We should do something different, or we shouldn't cover up the mistake that we did. That's moral courage. That's leadership at the individual level. It's also leadership at the institutional level where decisions are made. And again, the ability to see what it is critical in healthcare. Care being the critical piece. And the ability to keep that excellence in patient care as the primary factor that determines everything else. Not based on how much more money can I get if I put the patient through really unnecessary procedures but it's about what is in the best interest of the patient. So, keeping that care central again requires leadership. So what I'm saying is I think leadership must be authentic. And courageous at the same time. Institutionality if you look at the system there are these health policy decisions that we have to make. Who gets what? How much do you cover? How much do not cover? And again, it has to be courageous and authentic. When we look at the big picture of our populations, it is not about an individual. What was valid for the population may not work for individual right? And in the US in particular, it's been all about individual care and I think we need to really emphasize population based and preventive care. I am a primary care doctor. Preventive care so that we don't get into all of these illnesses, but wellness, health is what we need to protect as a community. So, I see leadership at all those levels”* |

´

Theme 5.0 Health, public health, and health systems and services.

| **Organization of the Coding** | **Interpretation/ “Important Quotes”** |
| --- | --- |
| 5. Theme: Health, public health, and health systems and services. | |
| Health Challenges | Perceived nation and across the Americas health, public health, and health systems and services challenges. |
| 5.1 Subtheme: Health workforce (HWF) and their education | |
| HWF and their education | Challenges related to HWF and how they are getting their education |
| Codes: HWF and their education | |
| Education crisis | Refers to an outgoing crisis in education in the Americas.  Participant #32: *“I see that medical education is going through a crisis, especially in Latin America. This is primarily because, since the Flexner report in the United States and Canada, our faculties have been heavily influenced by a purely scientific perspective and the widespread use of medications generalized in the population, programs have been developed based on separate subjects and departments, and in my country, there have been attempts to modify the curriculum to transition to an integrated model, which is uncommon in Latin America. Few universities in Latin America, to my knowledge, have successfully implemented an integrated curriculum with problem-based learning. However, this has led to various administrative issues. We still have people seeking validation of their titles, and with an integrated curriculum, it becomes complex and challenging to evaluate. In my faculty, the curriculum is course-based and still follows a structure of basic, preclinical, and clinical stages. I believe it's a critical moment for Latin America to rethink the type of professionals we aim to produce. We are going through a critical stage because, even though our curriculum is designed for general practitioners in primary care, there are limited job opportunities in primary care in my country. There are very few professional growth opportunities in primary care and it’s not considered important. There is little emphasis on it, and more than 95% of our graduates aspire to specialize as the next step, which is seen as a mandatory requirement to practice medicine. This is why I talk about a critical stage – it depends a lot on what medical schools do to find the true path for the ideal training of a medical professional. This begins with defining what we want to achieve in the undergraduate degree. That is where we are facing many challenges in reality, (unadible) in Colombia, where the implementation of these changes has only started to take place after 15 years, even after its inception, only in certain isolated implementation experiences. Therefore, we also have to consider whether we are going to train a doctor for that model of family, community, intercultural health. Because currently, as I tell you, the progression of our students through our career, and once they graduate, what they mostly seek is a specialization. And that obviously is in dissonance, it doesn't align with the profile of a primary care physician that we should supposedly be aiming for. The majority of our graduates are seeking specialization as the safest path for their professional practice and even for their personal development”*. |
| Strengthening primary care | The need to strengthen primary care and prioritize it within healthcare. |
| Transition to preventive medicine | The need to transition healthcare towards prevention and promotion. |
| Incompetent physicians | Physicians that manage to graduate without the necessary competencies. |
| Compartmentalization of education | Tendency to divide areas of knowledge within education and research |
| Effective screening | Refers to the need to educate students to conduct effective screening in patients and reduce mistakes |
| Fragmented care | Tendency in healthcare to treat patients in sub specializations resulting in fragmented care. |
| Research Literacy | Abilities related to scientific research: reading and writing. |
| Physician-patient relationship | The idea that the physician-patient relationship is crucial in healthcare and fostering well-being in patients. |
| Geographical location and infrastructure | The location of students along with the physical resources of the school influencing their education. |
| 5.2 Subtheme: Increase of communicable and non-communicable diseases &  situations that foster poorer health outcomes | |
| Increase of non-communicable and communicable diseases & situations that foster poorer health outcomes | An augmentation of diseases within the population along with specific situations and contextual conditions that foster poorer health outcomes |
| Codes: Increase of communicable and non-communicable disease & social challenges situations that foster poorer health outcomes | |
| Increase in oncological diseases | Oncological diseases becoming widespread and more common in our population. |
| Neurodegenerative diseases | Neurodegenerative diseases becoming more common. |
| Increase in chronic diseases | Chronic diseases becoming more common in our population.  Participant #2: *“We also face problems with access to healthcare for the population. There's often a lack of capacity for many of these diseases, and waiting lists are common”.* |
| Increase in Metaxenic diseases | Resurface of metaxenic diseases in the population |
| Oral health | Based on World Health Organization´s definition: [Oral health (who.int)](https://www.who.int/health-topics/oral-health)  Participant #29: “*In our field of dentistry, which I work in, one of the most prevalent diseases in the population is dental caries and periodontal diseases, which are inadequately covered by the national healthcare systems. I believe that, from what I see in the context of the Americas, there is a strong prevalence of chronic diseases that cannot be adequately managed by healthcare systems*”. |
| Epidemiological Stagnation | Refers to a prolonged period of limited or no progress in controlling the spread or impact of a disease within a population.  Participant #3:*“In Colombia, we experience what is known as an epidemiological stagnation. Why? Because we have a predominance of chronic diseases like hypertension and diabetes, mainly in the population cluster. These are the main diseases in Colombia. But when we break it down, we have regions like Chocó, La Guajira, the border area with Venezuela in Norte de Santander, where diseases that should have been overcome persist, along with high maternal mortality, child malnutrition, and infectious diseases. In addition to these, Colombia's geographical features expose us to vector-borne and tropical diseases”.* |
| Gun violence | Violence that arises due to the availability and accessibility guns. Typically, occurring in the USA, but also seen in other countries. Usually affecting children in schools but also healthcare workers in hospitals. |
| Opiod crisis | Effects of the over consumption of opioids on the population. |
| Truthful information and communication | Refers to the challenge within society of having access to truthful information and the ability to communicate it to them.  Participant #33: “*the very overarching challenge in US Healthcare currently, Is correct and truthful information and communication. Although the United States has many healthcare workers, we have plenty of money, we have great industries, the amount of misinformation that the public is getting, whether it's about vaccines, about prevention about control is at a time high, and is having a powerful negative effect on the health of the nation*”. |
| Armed conflict | Refers to a situation in which two or more parties engage in violent confrontation, typically involving the use of weapons, often for political, territorial, or ideological reasons.  Participant #3: “*Moreover, due to the social and economic conditions, we have diseases related to violence and the armed conflict. Considering all of this, the phenomenon of epidemiological stagnation is used to describe our situation. An example is the 12-year difference in life expectancy at birth between Bogotá and La Guajira, showing that environmental conditions significantly influence one's life prospects, regardless of healthcare services*”. |
| Migrant and refugees’ health | The health challenges of the migrant and refugees’ population |
| Shrinking population | Describes a demographic trend characterized by a decrease in the total number of individuals within a specific geographical area over time.  Participant #07: *“Also, those changes that other countries have experienced in terms of the inversion of the population pyramid we don't have them completely, but there are already projections for 2040, 2050, where we are going to see this population inversion, where there is going to be a larger adult population than children and all the problems that this entails, right? Obviously, with chronic degenerative diseases, which I am referring to: diabetes, hypertension, diseases such as renal insufficiency and one that the doctor was just mentioning a moment ago, depression”.* |
| Adverse Childhood Experiences populations’ health challenges | Groups of individuals who have been exposed to various forms of trauma, neglect, or abuse during their childhood, which can have long-term negative effects on their physical, emotional, and mental health outcomes |
| Technology advances | Refers to modern technology and how it is used to face health challenges.  Participant #14: *“Well, I believe that even at the global level we share health challenges. One of them is that health services, health care, the benefits of all the knowledge, technology, science, that has been developed in recent years or not, over the last decades, really reach the largest possible population. Because precisely in that analysis that was done in ANSER, back in 2010, what was seen is that all that progress that had occurred during the last century had only benefited a small part of the world's population. Let's say, there is an immense, profound inequity in terms of the benefits that are brought to the population through health systems and services. So, it seems to me that this is the first challenge in Colombia and in the region”.*  Participant#16: *“Okay, I feel that there are several challenges, at least from Peru, and I see what is often emphasized in Latin America is the existence of a precarious healthcare system. What does this mean? It means that we are lagging behind in considering all the new opportunities we have, such as technology and more. So, seeing that there are opportunities that we don't know how to take advantage of, I think one of the main challenges is that we don't harness that. If we're not utilizing everything as we should, such as the technology we have, it means that there are still hospitals in the healthcare system that manage medical records by hand or on paper, and they could transition everything to the technological/electronic side. Obviously, this requires training, many things in between, but it's a challenge that I think is considerable now, and I would also emphasize training in technology to improve healthcare”.* |
| Pandemics | outbreak of a contagious disease that spreads across multiple countries or continents, affecting a large number of people.  Participant #29: “*Health challenges. We are facing more pandemics due to climate change. We need to adapt. We are not prepared for these types of problems; there are many more pathologies associated with zoonoses. So, we need to prepare the healthcare system for that. Another problem is that there are several issues with vaccination systems, at least here in Mexico. We have almost eliminated our vaccination system. In Bogotá, it's slower. What else could be the challenges? Yes, I relate everything to climate change*”. |
| Covid-19 pandemic effects | Encompass a wide range of socio-economic, health-related, and psychological impacts, including disruptions to healthcare systems, economic downturns, social isolation, mental health challenges, and changes in behavior and lifestyle  Participant #22: *“I think that the pandemic and the resulting impacts, in terms of mental health, continue to be a huge issue”.* |
| Facing Covid | Refers to the mechanisms and systems established to combat the covid 19 pandemic.  Participant #33: “*Had the privilege to be on several global panels comparing different national challenges. Some countries, developed nations, didn't have the vaccine or the money to get it. Others would have the vaccine; they could purchase, or it was donated but they didn't have transportation to get it around. Others, didn’t have the health care workers. And as I said the US we've had the irony; we have all this wealth. We have all these things, but we have serious, serious miscommunication and we have unfortunately a substantial percentage of our population that is buying into various conspiracy theories and other problems that is actually against their well-being and best interests. So, these principles, that we’ve talked about so far today work at all levels. The level of which we're working, the psychosocial level is also: How we communicate that information*”. |
| 5.3 Subtheme: Climate Crisis | |
| The Climate Crisis | Effects on health (people’s, animals, plants, etc.) due to the climate crisis |
| Code: The Climate Crisis | |
| Health impacts caused by the climate crisis | The climate crisis effects on people’s, animal’s, plant’s, and the environment’s health. |
| 5.4 Theme: Social Inequality and the health systems | |
| Social Inequality and the health system | Health challenges due to the inequality latent on the Americas and within the health systems. It also relates to the strategies aiming to make the population healthy and keeping them healthy within the system.  Participant #28: “*So, it also seems to me that one of the great challenges is how to keep the population healthy. This involves health promotion and prevention, especially in certain chronic diseases that are challenging to control over an extended period and are economically costly for the healthcare system. Therefore, these functions, traditionally performed by the healthcare system, include promotion, prevention, care, rehabilitation, and disease control*”. |
| Codes: Social Inequality and the health systems | |
| Access and coverage | Refers to people’s access healthcare services and how these are distributed in a population. |
| Private vs. Public healthcare | The differences in a privatized vs. A public system of healthcare. |
| Lack of treatment access | Refers to the fact that even though disease treatment may be covered in the healthcare systems the actual treatment in reality faces restraints.  Participant #2: *“While these services are guaranteed, some people still have to wait because there are waiting lists, indicating inadequate access to these treatments. Medications are sometimes available, but the prompt access to treatment is lacking”.* |
| Waiting times and availability of treatment | Refers to difficulties arising from long waiting times and lack of availability of treatment in patients. |
| Inequity | Social inequalities affecting population health in Colombia and other countries.  Participant #3: *“Well, in my case as a public health expert, I have a connection with the Pan American Health Organization at the Colombia and regional level. So when you analyze health challenges, the Latin American region is one of the most inequitable regions in the world. It is the most inequitable region in the world by various indicators. Particularly, Colombia is considered the second or third most inequitable country in the world. This is reflected in health conditions, especially when other countries talk about demographic, epidemiological, and nutritional transitions. This contemplates that there is a shift from malnutrition to obesity and overweight, infectious diseases to chronic degenerative diseases, and cancer”.* |
| Limitation of resources | Resources of all sorts in healthcare being limited: financial, human, time, etc.  Participant #2: *“I think the challenges are related to resources. Resources in terms of human resources, economic resources, and infrastructure”.* |
| Healthcare a for profit business | Interpretation: Relates to the idea that the current healthcare system revolvers around making a profit and prioritizing economical gains instead of the well-being of people and their health.  *Participant #10 “There's another problem in the United States that I think is fairly unique amongst at least, you know, wealthier nations and that is that the health so-called health insurance business is also became nonprofit. I mean, I'm sorry it started is not nonprofit and became for profit and now they are in control of how doctors can practice medicine”.*  *Participant #20: “And then the second, I would say, is just the overall cost, or the amount of resources required for us to provide the health care that we're providing is immense, more than any other nation in the world and yet our outcomes are not as good as we would like to see. So, we're in a situation where we're spending a lot, we don't have the results that we would like to have for that high amount of expenditure. And it's very challenging then for people to pay for health insurance, for governments and companies to provide health insurance for their workers, because the costs are really high and the value is not equivalent to the costs in many cases. And then, as I said, if you look at people who are in the lower socioeconomic standing, their health care is particularly not what we would like it to be”.* |
| Health at the forefront | Refers to the need of prioritizing health within a society in comparison to other sectors in the economy.  Participant #14: *“And the second challenge precisely has to do with health being a discussion, or rather being the focus of interest of all the social and economic sectors of a country, a region, a territory. Health cannot be, be only focused on the health sector, on the health system, on the hospital sector, on the clinic, on quthe services. Health really comes into play; it is in the different areas where the development of human life occurs. At home, at work, in educational institutions, in places of recreation. And it is mediated by very important factors. In the health sector. In places that are outside, let's say, the government, the health authorities, such as the environment, the quality of housing, nutrition, whether the population has access to food, whether that food is healthy, anyway”*. |
| Sustainability of the system | Refers to the need to consider the sustainability of the healthcare system.  Participant #14: “*And there comes a fourth or fifth element, I already got lost in how many times I go, but it is also important to think about the sustainability of the system. Of health systems. But not only sustainability seen as a purely financial issue. Likewise, we see health systems that have a high allocation of resources from the State and society, but that are inefficient. So, sustainability must occur in a combination, where there is indeed adequate financing with some secure, solid resources, but also because the resources are well financed. And that they be invested from criteria of science, cost-effectiveness, but also, from criteria related to strengthening the issue of promoting, maintaining and strengthening the health of people who are not sick. These would be the challenges that I see as most relevant currently for the Colombian health system and for other similar countries in the region*”. |
| Health assurance/ keeping the public healthy | Health challenges related to strategies aiming to make the population healthy and keeping them healthy.  *Participant #28: “So, it also seems to me that one of the great challenges is how to keep the population healthy. This involves health promotion and prevention, especially in certain chronic diseases that are challenging to control over an extended period and are economically costly for the healthcare system. Therefore, these functions, traditionally performed by the healthcare system, include promotion, prevention, care, rehabilitation, and disease control”.* |
| An outdated healthcare system | Refers to the idea that the current health care system no longer responds to actual needs as it has become outdated.  Participant #26: “*Wow, that's an easy question to answer. She says sarcastically. Thank you. I think our system was designed for a time that is no longer. Relevant. And so, the systems we used, back then are not fit for today's purpose. So, I think this the struggle is or let me back up, it's sort of. Like you have, you know, a car and pieces just start breaking down on the car and so you replace the tires you replaced the engine you replaced you know the exhaust system and you just keep trying to piece it together and make it work but at the end of the day you're still You're now driving a car that's falling apart and been pieced back together and wasn't designed to fit purpose or what we really need to just do is say. This car needs to be put aside and We need to turn to, you know, an electric vehicle or whatever that happens to be that's more in tune with the needs of today. And so, I think the biggest. Prices or biggest challenge facing our system is that we don't have a system. And it works. That's fit for purpose. And different people are within the system. And they each see their part of the system. So, we think of it, you know, in that classic elephant in the room where everyone is looking at the same thing. Their perspective is different. And so, they're only seeing a piece of what they have. And in order to look at the system from a whole we need we need to take that step back so we can see the elephant for its full self to be able to address that. And so, from a leadership perspective, it's overwhelmingly daunting a task. To try to fit together when there are so many people, groups, organizations, governments. That are part of that situation. And so that's what I sort of see as the biggest challenges of us. That's all coming together. Putting our you know, differences aside and trying to focus on the key one and I'm not sure that's really realistically ever gonna happen, even in the most utopian of government situations*, *which we don't have in either Colombia or in Canada*”. |
| Little emphasis on health prevention and promotion | Current healthcare system that emphasizes the treatment of diseases over prevention and health promotion. |
| Plans driven by politics not experts | A problem that arises in healthcare when plans are driven by politics and not by experts in the manner. |
| Continuity of policy in the political arena | The idea that leadership in the healthcare system is influence by politics. |
| Social determinants of health & environmental determinants of health | Based on World Health Organization´s definitions. <https://www.who.int/health-topics/social-determinants-of-health> and [Environmental Determinants of Health - PAHO/WHO \| Pan American Health Organization](https://www.paho.org/en/topics/environmental-determinants-health)  Participant #22: “*The climate crisis, planetary justice, as well as equity access and participation in human rights, remain a global crisis, a public health crisis because of the impact. So whether that be food security, individuals experiencing homelessness, etcetera, in my world, and in pediatrics, the ACEs, or the adverse childhood events, all have an impact on health. And I think public health has a role to play for those”*.  Participant #21: “*I think there's a lot of challenges with the US health care system. I, unfortunately, can't speak to the Colombian health care system, but from what I know about living here locally and working in the emergency department, there's a huge problem with health care disparities and health outcomes at very significantly by socio-economic factors, race, gender, and sex, ethnicity. There's major issues with social determinants of health having a big impact on health outcomes. There's issues with people being able to access and afford preventive care that leads to a lot of use of the emergency department, which, you know, contributes to the high cost of our health care system. There's issues with educating patients to take ownership of their health and make educated decisions as health care consumers. As well as a lot of issues with costs associated with health care being opaque and difficult for people to understand and gain information about as they have decision making around their health. Yeah, I would say the biggest ones that come to mind is just inequities in the health care system and social determinants of health having huge impact on people's health care outcomes*”. |
| The health of the caretaker | Refers to the physical, mental, and emotional well-being of an individual who provides care and support to someone else, often a family member or friend, who is unable to care for themselves due to illness, disability, or other circumstances  Participant #14: *“Another important element is related to the people who attend to the health needs of other people, families, communities, and patients. And it is actually necessary that there be an adequate number of health personnel, colleagues, doctors, dentists, therapists, adequate to meet the needs of that population, because of course, we can have infrastructure, we can have financial resources, but if we do not have that number of adequately trained people, located in the appropriate sectors, because we are also going to continue with a deficit, particularly these people who already have some health problem, who need, let's say, with greater urgency of that type of care, these people trained, trained in health care. It would be a third challenge”*. |
| 5.5 Subtheme: Mental Health | |
| Mental Health | Health challenges related to mental health. |
| Codes: Mental Health | |
| Mental health | Mental health refers to a person's emotional, psychological, and social well-being, encompassing their ability to cope with stress, maintain positive relationships, and function effectively in all aspects of daily life.  Participant #2: *“Additionally, mental health problems have increased, especially postpandemic, as consultations were heavily postponed (...)”* |
| Limited focus on prevention in practice and training: mental health | The idea that in the medical syllabi to little emphasis is made on preventing and promoting mental health in the students themselves. |
