# Supplementary material for: The Meaning of Leadership in Medical Education in the Pan American Health Organization Member States: A Stakeholder Analysis and Interviews
Source: Int J Public Health. 2026 Feb 26;71:1608502. doi: 10.3389/ijph.2026.1608502 (PMC12979235; doi:10.3389/ijph.2026.1608502)
Supplement: Supplementary file 4 [file Supplementaryfile3.docx]

**Supplementary material 3. Mapping stakeholders in leadership education within undergraduate medical education in the Americas: a literature review with systematic components.**

Authors conducted a review to identify relevant stakeholders that work on the health and education system in undergraduate medical education. This review followed the Cochrane Systematic Review Version 6.3, 2022 [1]. The review question is framed according to the Population, Intervention, Comparators, Outcomes, and Settings (PICOS).

P: undergraduate medical students.

I: education.

C: comparator.

O: stakeholders.

S: The Americas.

A four-step search strategy was done. Firstly, an index literature was conducted in Medline via PubMed, Embase via Ovid, and LILACS (refer to Tables 1,2, and 3). This search was performed using three sets of terms: (i) population, which was focused on undergraduate medical education, (ii) the intervention, which was education and leadership, and (iii) settings directed to Colombia and the Americas. The hits from the databases were analyzed based on title, abstract, and full-text using Rayyan. Secondly, a search strategy was conducted in the official websites of the two ministries in Colombia (Ministry of Health and Social Protection and the Ministry of National Education). Thirdly, the first 100 results from a Google search were examined using the equation: leadership in medical education. Fourthly, we asked the stakeholders regarding potential actors to be interviewed and literature on leadership education that could be suitable for undergraduate education.

We read each piece of literature to determine if it was appropriate for this project (refer to Table 4). We did not perform quality assessment. From the literature, we extracted information including the authors, affiliations, and emails. If emails were not provided for each author; we looked at the professional webpage via LinkedIn and ORCID.

We identified 157 pieces of literature that cover at least one domain from the public health leadership competency framework model (refer to table 5). While we were sure that some of them covered leadership in undergraduate medical education, others focused on providing leadership education to the health workforce or the public health workforce. In such cases, we aimed to determine if leadership was specifically targeted at medical students. Nevertheless, we included this literature and reached out to the contact author via email to ask who was directed the leadership education.

References

1. Cochrane. Cochrane Handbook for Systematic Reviews of Interventions. <https://training.cochrane.org/handbook/current>

**Table 1. Search strategy: Medline via PubMED**

| Criteria. |  |
| --- | --- |
| Population | 1.Students, Medical [Mesh term].  2.Medical student* [ ti/ab].  3.Health profe* student* [ti,ab].  4.Public health profe* [ti,ab].  5.#1 OR # 2 OR # 3 OR # 4 OR #5 |
| Intervention | 6. Education, Medical [Mesh term]  7. Education, Public Health Professional [Mesh term]  8. undergraduate medical education [ti/ab]  9. medical education [ti/ab]  10. public health education [ti/ab]  11. Leadership[ti/ab]  12. Leadership[Mesh term]  13. #6 OR # 7 OR # 8 OR # 9 OR #10 OR #11 OR #12 |
| Outcomes | Not applicable |
| Comparator | Not applicable |
| Settings | 14. Colombia [Mesh term]  15. Americas [Mesh term]  16. Latin America and the Caribbean [ti/ab]  17. #14 OR # 15 OR # 16  18. #5 AND #13 AND #23  19. #18 AND Date of Publication 2010/01/01 to present |
| String | ((((((Students, Medical[MeSH Terms]) OR (Medical student*[Title/Abstract])) OR (Health profe* student*[Title/Abstract])) OR (Public health profe*[Title/Abstract])) AND (((((((Education, Medical[MeSH Terms]) OR (Education, Public Health Professional[MeSH Terms])) OR (undergraduate medical education[Title/Abstract])) OR (medical education[Title/Abstract])) OR (public health education[Title/Abstract])) OR (Leadership[Title/Abstract])) OR (Leadership[MeSH Terms]))) AND (((Colombia[MeSH Terms]) OR (Americas[MeSH Terms])) OR (Latin America[Title/Abstract] AND the Caribbean[Title/Abstract])) AND (2010:2023[pdat])) AND (("2010/01/01"[Date - Publication] : "3000"[Date - Publication])) |
| Hits  Date | 4520  January/ 12/ 2023 |

**Table 2. Search strategy: Embase via Ovid.**

| Criteria. |  |
| --- | --- |
| Population | 1.medical student [Subject Heading]  2.health student [subject heading]  3.Medical student* ti  4.Medical student* ab  5.health profe* student* ti.  6.health profe* student* ab.  7.public health profe* ti  8.public health profe* ab  9.#1 OR # 2 OR # 3 OR # 4 OR #5 OR # 6 OR #7 OR # 8 |
| Intervention | 10.medical education [ Subject Heading]  11.curriculum[ Subject Heading]  12.competency-based education ti  13.competency-based education.ab.  14.curriculum ti.  15.curriculum ab.  16.undergraduate medical education ti  17.undergraduate medical education ab  18. leadership all fields  19.#10 OR # 11 OR # 12 OR # 13 OR #14 OR # 15 OR #16 OR # 17 OR # 18  20. # 10 OR # 11 OR # 12 OR # 13 OR # 14 OR # 15 OR # 16 OR # 17 OR # 18 OR # 19 |
| Outcomes | Not applicable |
| Comparator | Not applicable |
| Settings | 21. Colombia [Subject Heading]  22. Western Hemisphere [Subject Heading]  23. Colombia [ti]  24. Colombia [ab]  25. Latin America and the Caribbean [ti]  26. Latin America and the Caribbean [ab]  27. #21OR # 22 OR # 23 OR # 24 OR# 25 OR # 26 |
|  | 28. #27 AND Publication year 2010- current |
| String | Embase <1974 to 2023 Week 01>  1 (medical student or health student).sh. or Medical student*.ti. or Medical student*.ab. or health profe* student*.ti. or health profe* student*.ab. or public health profe*.ab. 106693  2 (medical education or curriculum).sh. or competency-based education.ti. or competency-based education.ab. or curriculum.ti. or curriculum.ab. or undergraduate medical education.ti. or undergraduate medical education.ab. 330511  3 (colombia or Western Hemisphere).sh. or colombia.ti. or colombia.ab. or (Latin America and the Caribbean).ti. or (Latin America and the Caribbean).ab. 56382  4 1 and 2 and 3 102  5 limit 4 to yr="2010 -Current" 75 |
| Hits  Date | 75  12/ January/2023 |

**Table 3. Search strategy: LILACS via Portal Regional de la BVS - *Información y conocimiento para la salud.***

| Criteria. |  |
| --- | --- |
| Population | 1. Estudiantes [titulo, resumen, asunto] |
| Intervention | 1. Liderazgo [titulo, resumen, asunto] |
| Outcomes | Not applicable |
| Comparator | Not applicable |
| Settings | Not aplicable |
|  | (Estudiantes) AND (liderazgo)  Tick boxes:  Lenguage Español e Ingles  Intervalo de año de publicacion: 2010 2023 |
| Hits  Date | 70  12/01/2023 |

**Table 4. Eligibility criteria for stakeholders**

| Criteria | Inclusion | Exclusion |
| --- | --- | --- |
| Population | -Undergraduate Medical Education (UME).  - UME with a interprofessional and transprofesional education (IPE/ TPE) approach. | -Health workforce that does not cover undergraduate medical students.  - Public health that is not directed to undergraduate medical students |
| Intervention | -Stakeholders that desire to teach leadership* in UME+ IPE/TPE in the Americas.  - Stakeholders that teach leadership* in UME + IPE/TPE in the Americas.  -Stakeholders that desire to teach leadership* in UME+ IPE/TPE in Colombia.  - Stakeholders that teach leadership* in UME+ IPE/TPE in Colombia.  Leadership is conceptualised by the framework: Public Health Leadership Competency Framework Model. *At least one component to be included   1. System thinking 2. Political leadership 3. Leadership, organisational leadership and development 4. Leadership and communication 5. Leading change 6. Emotional intelligence and leadership in team-based organisations 7. Ethics and professionalism. | -Stakeholders that desire to teach leadership in UME, and they are located outside the Americas (No affiliation in the Americas)  - Stakeholders that are teaching leadership in UME, and they are located outside the Americas (No affiliation in the Americas)  - Stakeholders that use interchangeably leadership and management. |
| Outcomes | Actors that document the importance of leadership in UME in the Americas.  Actors that are teaching leadership in UME alone or with other professions/citizens. | Not applicable |
| Comparator | Not applicable | Not applicable |
| Settings | -Literature in Spanish and English.  - every study design. - ≥2010 (ASCOFAME documented that Leadership was important in Colombia) | -Literature in another language.  - Stakeholders  -≤2009 (ASCOFAME have not documented yet that Leadership was important in Colombia) |

**Table 5. Literature from the four steps for contacting stakeholders to be interviewed.**

| # | Literature |
| --- | --- |
| 1 | LeBlanc, C., Sonnenberg, L. K., King, S., & Busari, J. (2020). Medical education leadership: from diversity to inclusivity. *GMS journal for medical education*, *37*(2), Doc18. <https://doi.org/10.3205/zma001311> |
| 2 | Reyes B H. (2016). Acortar los estudios de pre título en Medicina, en Chile: ¿Ahora y para todos? [Shortening undergraduate medical training: now and for all medical schools in Chile?]. *Revista medica de Chile*, *144*(1), 7–10. https://doi.org/10.4067/S0034-98872016000100001 |
| 3 | Barry, E. S., Dong, T., Durning, S. J., Schreiber-Gregory, D., Torre, D., & Grunberg, N. E. (2019). Medical Student Leader Performance in an Applied Medical Field Practicum. *Military medicine*, *184*(11-12), 653–660. <https://doi.org/10.1093/milmed/usz121> |
| 4 | Barry, E. S., Grunberg, N. E., Kleber, H. G., McManigle, J. E., & Schoomaker, E. B. (2018). A four-year medical school leader and leadership education and development program. *International journal of medical education*, *9*, 99–100. https://doi.org/10.5116/ijme.5abe.12d2 |
| 5 | Reddy, R. M., Kim, A. W., Cooke, D. T., Yang, S. C., Vaporciyan, A., & Higgins, R. S. (2014). The looking to the future medical student program: recruiting tomorrow's leaders. *The Annals of thoracic surgery*, *97*(3), 741–743. <https://doi.org/10.1016/j.athoracsur.2013.09.117> |
| 6 | Oliveira, C. C., de Souza, R. C., Abe, E. H., Silva Móz, L. E., de Carvalho, L. R., & Domingues, M. A. (2014). Undergraduate research in medical education: a descriptive study of students' views. *BMC medical education*, *14*, 51. https://doi.org/10.1186/1472-6920-14-51 |
| 7 | Neeley, S. M., Clyne, B., & Resnick-Ault, D. (2017). The state of leadership education in US medical schools: results of a national survey. *Medical education online*, *22*(1), 1301697. https://doi.org/10.1080/10872981.2017.1301697 |
| 8 | Long, J. A., Lee, R. S., Federico, S., Battaglia, C., Wong, S., & Earnest, M. (2011). Developing leadership and advocacy skills in medical students through service learning. *Journal of public health management and practice : JPHMP*, *17*(4), 369–372. <https://doi.org/10.1097/PHH.0b013e3182140c47> |
| 9 | Clyne, B., Rapoza, B., & George, P. (2015). Leadership in Undergraduate Medical Education: Training Future Physician Leaders. *Rhode Island medical journal (2013)*, *98*(9), 36–40. |
| 10 | Matson, C. C., Lake, J. L., Bradshaw, R. D., & Matson, D. O. (2014). The public health leadership certificate: a public health and primary care interprofessional training opportunity. *Health promotion practice*, *15*(1 Suppl), 64S–70S. <https://doi.org/10.1177/1524839913509275> |
| 11 | Webb, A. M., Tsipis, N. E., McClellan, T. R., McNeil, M. J., Xu, M., Doty, J. P., & Taylor, D. C. (2014). A first step toward understanding best practices in leadership training in undergraduate medical education: a systematic review. *Academic medicine : journal of the Association of American Medical Colleges*, *89*(11), 1563–1570. <https://doi.org/10.1097/ACM.0000000000000502> |
| 12 | Ulloque, M. J., Villalba, S., Varela de Villalba, T., Fantini, A., Quinteros, S., & Díaz-Narváez, V. (2019). Empathy in medical students of Córdoba, Argentina. Niveles de empatía en estudiantes de medicina de Córdoba, Argentina. *Archivos argentinos de pediatria*, *117*(2), 81–86. https://doi.org/10.5546/aap.2019.eng.81 |
| 13 | Wayne, N. L., Vermillion, M., & Uijtdehaage, S. (2010). Gender differences in leadership amongst first-year medical students in the small-group setting. *Academic medicine : journal of the Association of American Medical Colleges*, *85*(8), 1276–1281. <https://doi.org/10.1097/ACM.0b013e3181e5f2ce> |
| 14 | Caron, R. M., Hiller, M. D., & Wyman, W. J. (2013). Engaging local public health system partnerships to educate the future public health workforce. *Journal of community health*, *38*(2), 268–276. <https://doi.org/10.1007/s10900-012-9610-8> |
| 15 | Matheus B. T. (2019). The important role of academic leagues (extensions) in Brazilian medical education. *Revista da Associacao Medica Brasileira (1992)*, *65*(2), 98–99. <https://doi.org/10.1590/1806-9282.65.2.98> |
| 16 | Idso, J. M., Helmen, Z. M., Hueston, W. J., & Meurer, J. R. (2019). Student Leadership Development Initiative: A Pilot for a Sustainable, Replicable Model for Incorporating Leadership into Medical Education. *WMJ : official publication of the State Medical Society of Wisconsin*, *118*(1), 39–41. |
| 17 | Richard, K., Noujaim, M., Thorndyke, L. E., & Fischer, M. A. (2019). Preparing Medical Students to Be Physician Leaders: A Leadership Training Program for Students Designed and Led by Students. *MedEdPORTAL : the journal of teaching and learning resources*, *15*, 10863. https://doi.org/10.15766/mep_2374-8265.10863 |
| 18 | Teo, A. R., Harleman, E., O'sullivan, P. S., & Maa, J. (2011). The key role of a transition course in preparing medical students for internship. *Academic medicine : journal of the Association of American Medical Colleges*, *86*(7), 860–865. <https://doi.org/10.1097/ACM.0b013e31821d6ae2> |
| 19 | Sheline, B., Tran, A. N., Jackson, J., Peyser, B., Rogers, S., & Engle, D. (2014). The Primary Care Leadership Track at the Duke University School of Medicine: creating change agents to improve population health. *Academic medicine : journal of the Association of American Medical Colleges*, *89*(10), 1370–1374. <https://doi.org/10.1097/ACM.0000000000000305> |
| 20 | Girotti, J. A., Loy, G. L., Michel, J. L., & Henderson, V. A. (2015). The Urban Medicine Program: Developing Physician-Leaders to Serve Underserved Urban Communities. *Academic medicine : journal of the Association of American Medical Colleges*, *90*(12), 1658–1666. <https://doi.org/10.1097/ACM.0000000000000970> |
| 21 | Coleman, M. M., Blatt, B., & Greenberg, L. (2012). Preparing students to be academicians: a national student-led summer program in teaching, leadership, scholarship, and academic medical career-building. *Academic medicine : journal of the Association of American Medical Colleges*, *87*(12), 1734–1741. <https://doi.org/10.1097/ACM.0b013e318271cfd6> |
| 22 | Baker, S., & Daginawala, N. (2011). Leadership training for radiologists: a survey of opportunities and participants in MBA and MPH programs by medical students, residents, and current chairpersons. *Journal of the American College of Radiology : JACR*, *8*(8), 563–567. <https://doi.org/10.1016/j.jacr.2011.02.013> |
| 23 | Martins, A. C., Oliveira, F. R., Delfino, B. M., Pereira, T. M., de Moraes, F. H., Barbosa, G. V., de Macedo, L. F., Domingos, T.daS., Da Silva, D. P., Menezes, C. C., Oliveira Filho, E. S., Pereira, T. A., Piccirilli, E. S., & Pinto, W.deJ. (2015). How we enhanced medical academics skills and reduced social inequities using an academic teaching program. *Medical teacher*, *37*(11), 1003–1007. <https://doi.org/10.3109/0142159X.2014.970618> |
| 24 | Cadieux, D. C., Lingard, L., Kwiatkowski, D., Van Deven, T., Bryant, M., & Tithecott, G. (2017). Challenges in Translation: Lessons from Using Business Pedagogy to Teach Leadership in Undergraduate Medicine. *Teaching and learning in medicine*, *29*(2), 207–215. <https://doi.org/10.1080/10401334.2016.1237361> |
| 25 | Arroliga, A. C., Huber, C., Myers, J. D., Dieckert, J. P., & Wesson, D. (2014). Leadership in health care for the 21st century: challenges and opportunities. *The American journal of medicine*, *127*(3), 246–249. <https://doi.org/10.1016/j.amjmed.2013.11.004> |
| 26 | De Oliveira, D. F. M., Simas, B. C. C., Guimarães Caldeira, A. L., Medeiros, A. G. E. B., Freitas, M. R., Diniz, J., Jr, & Diniz, R. (2018). School of Medicine of Federal University of Rio Grande Do Norte: A traditional curriculum with innovative trends in medical education. *Medical teacher*, *40*(5), 467–471. <https://doi.org/10.1080/0142159X.2018.1440080> |
| 27 | Soriano-Moreno, A. N., Delgado-Raygada, J. E., Peralta, C. I., Serrano-Díaz, E. S., Canaza-Apaza, J. M., & Toro-Huamanchumo, C. J. (2020). Perception of medical students about courses based on peer-assisted learning in five Peruvian universities. *BMC research notes*, *13*(1), 391. <https://doi.org/10.1186/s13104-020-05237-5> |
| 28 | Chen, H. C., Wamsley, M. A., Azzam, A., Julian, K., Irby, D. M., & O'Sullivan, P. S. (2017). The Health Professions Education Pathway: Preparing Students, Residents, and Fellows to Become Future Educators. *Teaching and learning in medicine*, *29*(2), 216–227. <https://doi.org/10.1080/10401334.2016.1230500> |
| 29 | Alfaro-Toloza, P., & Olmos-de-Aguilera, R. (2013). Medical research and students in Latin America. *Lancet (London, England)*, *382*(9904), 1553. <https://doi.org/10.1016/S0140-6736(13)62324-7> |
| 30 | Ortiz-Moreira, L. E., Fasce-Henry, E. A., Matus-Betancourt, O. B., & Campos C, I. (2018). Educación médica y diversidad cultural en Chile. Un diálogo pendiente [Medical education and cultural diversity in Chile. A pending dialogue]. *Revista medica de Chile*, *146*(7), 922–928. <https://doi.org/10.4067/s0034-98872018000700922> |
| 31 | Málaga, G., Gayoso, D., & Vásquez, N. (2020). Empathy in medical students of a private university in Lima, Peru: A descriptive study. Empatía en estudiantes de medicina de una universidad privada de Lima, Perú: estudio descriptivo. *Medwave*, *20*(4), e7905. https://doi.org/10.5867/medwave.2020.04.7905 |
| 32 | Centeno, A., Ortiz, L., Campos, S., & Matus, O. (2016). South American investigation on professionalism: a theoretical framework. *Medical education*, *50*(11), 1153. <https://doi.org/10.1111/medu.13181> |
| 33 | Böhm, P., Soffia, A., Díaz, L. A., Villagrán, I., Pizarro, M., Collins, L. M., Riquelme, A., & Monrroy, H. (2020). Innovación docente: catorce años de experiencia en un curso teórico de pregrado en medicina [Teaching innovation in an undergraduate theoretical medicine course]. *Revista medica de Chile*, *148*(11), 1659–1667. <https://doi.org/10.4067/S0034-98872020001101659> |
| 34 | Youssef, F. F., Nunes, P., Sa, B., & Williams, S. (2014). An exploration of changes in cognitive and emotional empathy among medical students in the Caribbean. *International journal of medical education*, *5*, 185–192. <https://doi.org/10.5116/ijme.5412.e641> |
| 35 | Reyes, D., Isbej, L., Uribe, J., Ruz, C., Pizarro, M., Walker, R., Pérez-Cruz, P., Maldonado, A., Robles, C., Latorre, G., Ivanovic-Zuvic, D., Figueroa, C., González, A., Cotoras, P., Núñez, C., Labarca, J., & Riquelme, A. (2019). Portafolio en pregrado de Medicina: impacto educacional a 10 años de su implementación [Educational impact after 10 years of implementation of a portfolio for undergraduate medical students]. *Revista medica de Chile*, *147*(6), 790–798. <https://doi.org/10.4067/S0034-98872019000600790> |
| 36 | Schweller, M., Costa, F. O., Antônio, M. Â., Amaral, E. M., & de Carvalho-Filho, M. A. (2014). The impact of simulated medical consultations on the empathy levels of students at one medical school. *Academic medicine : journal of the Association of American Medical Colleges*, *89*(4), 632–637. <https://doi.org/10.1097/ACM.0000000000000175> |
| 37 | Tsao, P., & Yu, C. H. (2016). " There's no billing code for empathy " - Animated comics remind medical students of empathy: a qualitative study. *BMC medical education*, *16*(1), 204. <https://doi.org/10.1186/s12909-016-0724-z> |
| 38 | Belkowitz, J., Sanders, L. M., Zhang, C., Agarwal, G., Lichtstein, D., Mechaber, A. J., & Chung, E. K. (2014). Teaching health advocacy to medical students: a comparison study. *Journal of public health management and practice : JPHMP*, *20*(6), E10–E19. <https://doi.org/10.1097/PHH.0000000000000031> |
| 39 | McCloskey, L., Condon, R., Shanahan, C. W., Wolff, J., Culler, C., & Kalish, R. (2011). Public health, medicine, and dentistry as partners in community health: a pioneering initiative in interprofessional, practice-based education. *Journal of public health management and practice : JPHMP*, *17*(4), 298–307. <https://doi.org/10.1097/PHH.0b013e3182165013> |
| 40 | Costa, P., de Carvalho-Filho, M. A., Schweller, M., Thiemann, P., Salgueira, A., Benson, J., Costa, M. J., & Quince, T. (2017). Measuring Medical Students' Empathy: Exploring the Underlying Constructs of and Associations Between Two Widely Used Self-Report Instruments in Five Countries. *Academic medicine : journal of the Association of American Medical Colleges*, *92*(6), 860–867. <https://doi.org/10.1097/ACM.0000000000001449> |
| 41 | Franco, C. A. G. D. S., Franco, R. S., Lopes, J. M. C., Severo, M., & Ferreira, M. A. (2018). Clinical communication skills and professionalism education are required from the beginning of medical training - a point of view of family physicians. *BMC medical education*, *18*(1), 43. <https://doi.org/10.1186/s12909-018-1141-2> |
| 42 | Rotenstein, L., Perez, K., Wohler, D., Sanders, S., Im, D., Kazberouk, A., & Phillips, R. S. (2019). Preparing health professions students to lead change. *Leadership in health services (Bradford, England)*, *32*(2), 182–194. <https://doi.org/10.1108/LHS-02-2018-0011> |
| 43 | Rodríguez-Villamizar, L. A., Vera-Cala, L. M., Rojas-Sánchez, O. A., Rivera-Carvajal, R., & Uribe-Rivero, L. M. (2017). Evaluación de contenidos curriculares y percepción de competencias de estudiantes del área de la salud respecto del Modelo Integral de Atención en Salud en Colombia [Assessment of curricular contents and perception of competencies of health students related to the integrated health care model in Colombia]. *Revista de salud publica (Bogota, Colombia)*, *19*(4), 491–498. <https://doi.org/10.15446/rsap.v19n4.67261> |
| 44 | Gruner, D., Feinberg, Y., Venables, M. J., Shanza Hashmi, S., Saad, A., Archibald, D., & Pottie, K. (2022). An undergraduate medical education framework for refugee and migrant health: Curriculum development and conceptual approaches. *BMC medical education*, *22*(1), 374. <https://doi.org/10.1186/s12909-022-03413-8> |
| 45 | Hamui-Sutton, A., Varela-Ruiz, M., Ortiz-Montalvo, A., & Torruco-García, U. (2015). Modelo educativo para desarrollar actividades profesionales confiables (MEDAPROC) [Educational model to develop trustworthy professional activities]. *Revista medica del Instituto Mexicano del Seguro Social*, *53*(5), 616–629. |
| 46 | MacKechnie, M. C., Miclau, T. A., Cordero, D. M., Tahir, P., & Miclau, T., 3rd (2022). Leadership development programs for healthcare professionals in low-and middle-income countries: A systematic review. *The International journal of health planning and management*, *37*(4), 2149–2166. <https://doi.org/10.1002/hpm.3457> |
| 47 | Ament Giuliani Franco, C., Franco, R. S., Cecilio-Fernandes, D., Severo, M., Ferreira, M. A., & de Carvalho-Filho, M. A. (2020). Added value of assessing medical students' reflective writings in communication skills training: a longitudinal study in four academic centres. *BMJ open*, *10*(11), e038898. <https://doi.org/10.1136/bmjopen-2020-038898> |
| 48 | Davalos-Batallas, V., Vargas-Martínez, A. M., Bonilla-Sierra, P., Leon-Larios, F., Lomas-Campos, M. D., Vaca-Gallegos, S. L., & de Diego-Cordero, R. (2020). Compassionate Engagement and Action in the Education for Health Care Professions: A Cross-Sectional Study at an Ecuadorian University. *International journal of environmental research and public health*, *17*(15), 5425. <https://doi.org/10.3390/ijerph17155425> |
| 49 | Hernández-Rincón, E. H., Pimentel-González, J. P., Orozco-Beltrán, D., & Carratalá-Munuera, C. (2016). Inclusion of the equity focus and social determinants of health in health care education programmes in Colombia: a qualitative approach. *Family practice*, *33*(3), 268–273. <https://doi.org/10.1093/fampra/cmw010> |
| 50 | Abreu-Oliveira, Mayara de, Rangel-Rosa, Kamila, Rezende-Dázio, Eliza Maria, Castro-Ribeiro, Anderson de, & Silva-Lima, Rogério. (2022). Aprendizaje de competencias no técnicas en las urgencias prehospitalarias: perspectiva de los estudiantes de Medicina. *Revista Médica Electrónica*, *44*(4), 700-713. Epub 31 de agosto de 2022. Recuperado en 21 de agosto de 2024, de <http://scielo.sld.cu/scielo.php?script=sci_arttext&pid=S1684-18242022000400700&lng=es&tlng=es>. |
| 51 | García, Héctor Atilio, Arce, Maura, Hoberuk, Tania, Cañete, Felicia, & Szwako, Andrés. (2021). Competencias transversales en los tres primeros años de la carrera de medicina malla 2015: una aproximación desde la percepción de los actores clave. *Anales de la Facultad de Ciencias Médicas (Asunción)*, *54*(3), 67-84. Epub December 00, 2021.<https://doi.org/10.18004/anales/2021.054.03.67> |
| 52 | Martinez Ozuna, Gregorio, Santos Guzmán, Jesús, Beltran, Eduardo Leal, & Hernandez Angeles, Angélica Lizeth. (2020). Liderazgo transformacional en estudiantes de ciencias de la salud. *Educación Médica Superior*, *34*(2), . Epub 01 de junio de 2020. Recuperado en 21 de agosto de 2024, de <http://scielo.sld.cu/scielo.php?script=sci_arttext&pid=S0864-21412020000200007&lng=es&tlng=es>. |
| 53 | Cajachagua Castro, Mayela, Mamani Limachi, Rut, Miranda Limachi, Keila, & Davila Villavicencio, Roussel. (2020). Educación interprofesional y vivencias de los estudiantes en la ejecución de proyectos de desarrollo social. *Revista Cubana de Enfermería*, *36*(2), . Epub 01 de junio de 2020. Recuperado en 21 de agosto de 2024, de <http://scielo.sld.cu/scielo.php?script=sci_arttext&pid=S0864-03192020000200005&lng=es&tlng=es>. |
| 54 | Rolle, A. J., Lubi, F., & Ramirez, M. (2016). Voluntariado en medicina. Proyecciones asociadas a su desarrollo. *ARS MEDICA Revista De Ciencias Médicas*, *41*(3), 34–37. <https://doi.org/10.11565/arsmed.v41i3.372> |
| 55 | Bustamante, E. & Sanabria, Álvaro. ( 2014). Evaluación de las actitudes hacia el profesionalismo en estudiantes de medicina. *Revista Colombiana de Cirugía*, 29 (3), 222–229. DOI:https://doi.org/10.30944/20117582.404. |
| 56 | Jorge, M. L., Coelho, I. C., Paraizo, M. M., & Paciornik, E. F. (2014). Leadership, management and teamwork learning through an extra-curricular project for medical students: descriptive study. *Sao Paulo medical journal = Revista paulista de medicina*, *132*(5), 303–306. <https://doi.org/10.1590/1516-3180.2014.1325685> |
| 57 | Galindo Cárdenas LA, Arango Rave ME, Díaz Hernández DP, Villegas Múnera EM, Aguirre Muñoz CE, Kambourova M, Jaramillo Marín PA. ¿Cómo el aprendizaje basado en problemas (ABP) transforma los sentidos educativos del programa de Medicina de la Universidad de Antioquia?. Iatreia [Internet]. 6 de febrero de 2012 [citado 21 de agosto de 2024];24(3):Pág. 325-334. Disponible en: <https://revistas.udea.edu.co/index.php/iatreia/article/view/10553> |
| 58 | SANTAMARÍA, C., & SÁNCHEZ, M. O. (2011). Participación y liderazgo estudiantil: Una apuesta por la ciudadanía activa. *Colombia Médica, 42*(2), 103-112. |
| 59 | Gobierno de Colombia: Ministerio de Salud. Perfiles y competencias profesionales en salud. Available from: <https://www.minsalud.gov.co/sites/rid/Lists/BibliotecaDigital/RIDE/VS/TH/Perfiles-profesionales-salud.pdf> Accessed on April 6th 2020 |
| 60 | Gobierno de Colombia: Ministerio de Salud. Política Nacional de Talento Humano en Salud Dirección de Desarrollo del Talento Humano en Salud. Available from <https://www.minsalud.gov.co/sites/rid/Lists/BibliotecaDigital/RIDE/VS/TH/politica-nacional-talento-humano-salud.pdf> Accessed on April 6th 2020 |
| 61 | Gobierno de Colombia: Ministerio de Salud. Documento de recomendaciones para la transformación de la educación médica en Colombia. Available from: [Documento de recomendaciones para la transformación de la educación médica en Colombia (unisabana.edu.co)](https://intellectum.unisabana.edu.co/handle/10818/32984) Accessed on April 6th 2020 |
| 62 | Organismo Andino de Salud – Convenio Hipólito Unanue. Componente de Educación Permanente en Salud de la Política y Plan Andino de Recursos Humanos para la Salud; Lima: ORAS-CONHU, 2018. Available from: [Componente de educación permanente de la Política y Plan Andino de Recursos Humanos para la Salud del Organismo Andino de Salud \| Lima; Organismo Andino de Salud Convenio Hipólito Unanue; 2019; 2019. 19 p. \| LILACS \| LIPECS \| RHS (bvsalud.org)](https://pesquisa.bvsalud.org/portal/resource/pt/biblio-1148216) Accessed on April 6th 2020 |
| 63 | Puertas, E. B., Sotelo, J. M., & Ramos, G. (2020). Liderazgo y gestión estratégica en sistemas de salud basados en atención primaria de salud [Leadership and strategic management in health systems based on primary health care]. *Revista panamericana de salud publica = Pan American journal of public health*, *44*, e124. <https://doi.org/10.26633/RPSP.2020.124> |
| 64 | Campbell Barr, E., & Marmot, M. (2021). Liderazgo, determinantes sociales de la salud y equidad en la salud: el caso de Costa Rica [Leadership, social determinants of health and health equity: the case of Costa Rica]. *Revista panamericana de salud publica = Pan American journal of public health*, *45*, e101. <https://doi.org/10.26633/RPSP.2021.101> |
| 65 | Organizacion Panamericana de la Salud. Curso Liderazgo para la Gestión de las Políticas, la Regulación y la Planificación de los Recursos Humanos en Salud – 2022. Available from: [Curso Liderazgo para la Gestión de las Políticas, la Regulación y la Planificación de los Recursos Humanos en Salud – 2022 \| Campus Virtual de Salud Pública (CVSP/OPS) (paho.org)](https://campus.paho.org/es/curso/curso-liderazgo-para-la-gestion-de-las-politicas-la-regulacion-y-la-planificacion-de-los) Accessed on April 6th 2020 |
| 66 | Hernández Gracia, Tirso Javier, Duana Avila, Danae, & Polo Jiménez, Sergio Demetrio. (2021). Clima organizacional y liderazgo en un instituto de salud pública mexicano. *Revista Cubana de Salud Pública*, *47*(2), . Epub 30 de junio de 2021. Recuperado en 21 de agosto de 2024, de <http://scielo.sld.cu/scielo.php?script=sci_arttext&pid=S0864-34662021000200010&lng=es&tlng=es>. |
| 67 | Organizacion Panamericana de la salud. Programa de pasantías de la OPS - Fomentar el liderazgo futuro en la salud pública. Available from: [Programa de pasantías de la OPS - Fomentar el liderazgo futuro en la salud pública - OPS/OMS \| Organización Panamericana de la Salud (paho.org)](https://www.paho.org/es/carreras-ops/programa-pasantias-ops-fomentar-liderazgo-futuro-salud-publica) Accessed on April 6th 2020 |
| 68 | Organizacion Panamericana de la salud. Profesionales de la salud buscan ampliar conocimientos y capacidades para liderazgo en salud internacional. Available from: [Profesionales de la salud buscan ampliar conocimientos y capacidades para liderazgo en salud internacional \| Campus Virtual de Salud Pública (CVSP/OPS) (paho.org)](https://campus.paho.org/es/profesionales-de-la-salud-buscan-ampliar-conocimientos-y-capacidades-para-liderazgo-en-salud) Accessed on April 6th 2020 |
| 69 | Organizacion Panamerinaca de la salud. Liderazgo del sector salud de Paraguay para enfrentar la pandemia en el 2020 y una coyuntura para agilizar la reforma del sector con la cooperación técnica de la OPS/OMS. Available from: [Liderazgo del sector salud de Paraguay para enfrentar la pandemia en el 2020 y una coyuntura para agilizar la reforma del sector con la cooperación técnica de la OPS/OMS - OPS/OMS \| Organización Panamericana de la Salud (paho.org)](https://www.paho.org/es/documentos/liderazgo-sector-salud-paraguay-para-enfrentar-pandemia-2020-coyuntura-para-agilizar) Accessed on April 6th 2020 |
| 70 | Flores-Domínguez, Carmina. (2012). Feminización en medicina: liderazgo y academia. *Educación Médica*, *15*(4), 191-195. Recuperado en 21 de agosto de 2024, de <http://scielo.isciii.es/scielo.php?script=sci_arttext&pid=S1575-18132012000400003&lng=es&tlng=es>. |
| 71 | Sánchez Mendiola, Melchor. (2015). Liderazgo en medicina: ¿debemos enseñarlo y evaluarlo?. *Investigación en educación médica*, *4*(14), 99-107. Recuperado en 21 de agosto de 2024, de <http://www.scielo.org.mx/scielo.php?script=sci_arttext&pid=S2007-50572015000200008&lng=es&tlng=es>. |
| 72 | Aguirre-Gas HG & Mazón-González B. Calidad y liderazgo en medicina .2013. Available from: <https://biblat.unam.mx/hevila/RevistaCONAMED/2013/vol18/no4/4.pdf> Accessed on April 6th 2020 |
| 73 | Colegio Medico de Chile. Liderazgo en el equipo sanitaria.2019. Available from: [Liderazgo en el equipo sanitario \| Colegio Medico de Chile](https://revista.colegiomedico.cl/liderazgo-en-el-equipo-sanitario/) Accessed on April 6th 2020 |
| 74 | Llaque Dávila Wálter. Liderazgo - Ética Médica. 2011. Available from: <https://anmperu.org.pe/anales/2011/liderazgo_etica_medica.pdf> Accessed on April 6th 2020 |
| 75 | Abreu Cervantes, Arleen, & Téllez Cabrera, Maritza Yuliet. (2018). Líderes universitarios y protagonismo estudiantil. Caso Universidad de Ciencias Médicas de Camagüey. *Humanidades Médicas*, *18*(3), 504-520. Recuperado en 21 de agosto de 2024, de <http://scielo.sld.cu/scielo.php?script=sci_arttext&pid=S1727-81202018000300504&lng=es&tlng=es> |
| 76 | Frenk, J., Chen, L., Bhutta, Z. A., Cohen, J., Crisp, N., Evans, T., Fineberg, H., Garcia, P., Ke, Y., Kelley, P., Kistnasamy, B., Meleis, A., Naylor, D., Pablos-Mendez, A., Reddy, S., Scrimshaw, S., Sepulveda, J., Serwadda, D., & Zurayk, H. (2010). Health professionals for a new century: transforming education to strengthen health systems in an interdependent world. *Lancet (London, England)*, *376*(9756), 1923–1958. <https://doi.org/10.1016/S0140-6736(10)61854-5> |
| 77 | Frenk, J., Chen, L. C., Chandran, L., Groff, E. O. H., King, R., Meleis, A., & Fineberg, H. V. (2022). Challenges and opportunities for educating health professionals after the COVID-19 pandemic. *Lancet (London, England)*, *400*(10362), 1539–1556. <https://doi.org/10.1016/S0140-6736(22)02092-X> |
| 78 | Trujillo Condes VE, Bocanegra Lozano PD, Jiménez Garcés C, Henández González MM, Domínguez del Pino TM & Vieyra Reyes P. La competencia de liderazgo como elemento de la formación integral del medico. 2022. Available from: <http://ri.uaemex.mx/handle/20.500.11799/137912> Accessed on April 6th 2020 |
| 79 | Veranes Garzón, Inerkys, Peñalver Sinclay, Ana Gladys, & Jorna Calixto, Ana Rosa. (2021). Liderazgo en salud, motivación e inteligencia emocional. *Infodir*, (34), . Epub 01 de abril de 2021. Recuperado en 21 de agosto de 2024, de <http://scielo.sld.cu/scielo.php?script=sci_arttext&pid=S1996-35212021000100011&lng=es&tlng=es>. |
| 80 | Campos-Sánchez, C., Acosta-Escanaverino, I., Guillen-León, L., Rodríguez-Hurtado, D., Bouza-García, L., & Rodríguez-Pérez, A. (2022). Liderazgo en estudiantes de nuevo ingreso a las ciencias médicas. *EsTuSalud, 4*(1), e127. Recuperado de <https://revestusalud.sld.cu/index.php/estusalud/article/view/127/127> |
| 81 | Bandeira, I. D., & Mendoza, J. (2018). Medical education and leadership: a call to action for Brazil's mental health system. *International journal of medical education*, *9*, 170–172. <https://doi.org/10.5116/ijme.5b1b.b0a2> |
| 82 | Bernard, A., Ortiz, S. C., Jones, E., Heung, M., Guetterman, T. C. ., & Kirst, N. (2022). The Pandemic Leadership Model: A Study of Medical Student Values During COVID-19. *International Journal of Medical Students*, *9*(4), 274–281. https://doi.org/10.5195/ijms.2021.1001 (Original work published December 16, 2021) |
| 83 | Hashmi, S. S., Saad, A., Leps, C., Gillies-Podgorecki, J., Feeney, B., Hardy, C., Falzone, N., Archibald, D., Hoang, T., Bond, A., Wang, J., Alkhateeb, Q., Penney, D., DiFalco, A., & Pottie, K. (2020). A student-led curriculum framework for homeless and vulnerably housed populations. *BMC medical education*, *20*(1), 232. <https://doi.org/10.1186/s12909-020-02143-z> |
| 84 | Rajeh, N., Grant, J., Farsi, J., & Tekian, A. (2020). Contextual Analysis of Stakeholder Opinion on Management and Leadership Competencies for Undergraduate Medical Education: Informing Course Design. *Journal of medical education and curricular development*, *7*, 2382120520948866. <https://doi.org/10.1177/2382120520948866> |
| 85 | Portney, D. S., VonAchen, P., Standiford, T., Carey, M. R., Vu, J., Kirst, N., & Zink, B. (2019). Medical Student Consulting: Providing Students Leadership and Business Opportunities While Positively Impacting the Community. *MedEdPORTAL : the journal of teaching and learning resources*, *15*, 10838. <https://doi.org/10.15766/mep_2374-8265.10838> |
| 86 | Wagenschutz, H., McKean, E. L., Mangrulkar, R., Zurales, K., & Santen, S. (2019). A first-year leadership programme for medical students. *The clinical teacher*, *16*(6), 623–629. <https://doi.org/10.1111/tct.13005> |
| 87 | Mangrulkar, R. S., Tsai, A., Cox, S. M., Halaas, G. W., Nelson, E. A., Nesse, R. E., Silvestri, R. C., Radabaugh, C. L., Skochelak, S., Beck Dallaghan, G. L., & Steiner, B. (2020). A Proposed Shared Vision for Leadership Development for all Medical Students: A Call from a Coalition of Diverse Medical Schools. *Teaching and learning in medicine*, *32*(5), 561–568. <https://doi.org/10.1080/10401334.2020.1754835> |
| 88 | Dickerman, J., Sánchez, J. P., Portela-Martinez, M., & Roldan, E. (2018). Leadership and Academic Medicine: Preparing Medical Students and Residents to Be Effective Leaders for the 21st Century. *MedEdPORTAL : the journal of teaching and learning resources*, *14*, 10677. <https://doi.org/10.15766/mep_2374-8265.10677> |
| 89 | Mullan, P. B., Williams, J., Malani, P. N., Riba, M., Haig, A., Perry, J., Kolars, J. C., Mangrulkar, R., & Williams, B. (2014). Promoting medical students' reflection on competencies to advance a global health equities curriculum. *BMC medical education*, *14*, 91. <https://doi.org/10.1186/1472-6920-14-91> |
| 90 | Warde, C. M., Vermillion, M., & Uijtdehaage, S. (2014). A medical student leadership course led to teamwork, advocacy, and mindfulness. *Family medicine*, *46*(6), 459–462. |
| 91 | Varkey, P., Peloquin, J., Reed, D., Lindor, K., & Harris, I. (2009). Leadership curriculum in undergraduate medical education: a study of student and faculty perspectives. *Medical teacher*, *31*(3), 244–250. <https://doi.org/10.1080/01421590802144278> |
| 92 | Thibault G. E. (2020). The future of health professions education: Emerging trends in the United States. *FASEB bioAdvances*, *2*(12), 685–694. <https://doi.org/10.1096/fba.2020-00061> |
| 93 | Yphantides, N., Escoboza, S., & Macchione, N. (2015). Leadership in public health: new competencies for the future. *Frontiers in public health*, *3*, 24. <https://doi.org/10.3389/fpubh.2015.00024> |
| 94 | James, E., Evans, M., & Mi, M. (2021). Leadership Training and Undergraduate Medical Education: a Scoping Review. *Medical science educator*, *31*(4), 1501–1509. https://doi.org/10.1007/s40670-021-01308-9 |
| 95 | Álvarez García F & Casas Orozco MP. Relación entre el liderazgo del talento humano y la atención al usuario en los servicios de salud. 2022. Available from: <https://bibliotecadigital.udea.edu.co/bitstream/10495/32668/1/CasasMaria_2022_RelacionLiderazgoAtencionensalud.pdf> Accessed on April 6th 2020 |
| 96 | Pinzón Espitia O. Liderazgo en los servicios de salud. 2014. Available from: <https://www.google.com/url?sa=t&rct=j&q=&esrc=s&source=web&cd=&cad=rja&uact=8&ved=2ahUKEwi2gpO314WIAxWG_7sIHVkVKEsQFnoECBcQAQ&url=https%3A%2F%2Fdialnet.unirioja.es%2Fservlet%2Farticulo%3Fcodigo%3D6635354&usg=AOvVaw2wkyBxZG21qwQ5OrJvHXw5&opi=89978449> Accessed on April 6th 2020 |
| 97 | Gobierno de Colombia. Plan estrategico del talento humano.2020. Available from: <https://www.minambiente.gov.co/wp-content/uploads/2022/01/PLAN_ESTRATEGICO_DE_TALENTO_HUMANO.pdf> Accessed on April 6th 2020 |
| 98 | The Association of Schools and Programs of Public Health. Climate Change and Health A Public Health Education Toolkit. 2022. Available from: <https://s3.amazonaws.com/ASPPH_Media_Files/Climate_Change_and_Health_Education_Toolkit.pdf> Accessed on April 6th 2020 |
| 99 | Mintz, L. J., & Stoller, J. K. (2014). A systematic review of physician leadership and emotional intelligence. *Journal of graduate medical education*, *6*(1), 21–31. https://doi.org/10.4300/JGME-D-13-00012.1 |
| 100 | Chao, C., Wooten, K., Spratt, H., Sarraj, H., Aronson, J., Hommel, J., Ungerleider, R., Ungerleider, J. D., & Hellmich, M. R. (2018). Integration of leadership training for graduate and medical students engaged in translational biomedical research: Examining self-efficacy and self-insight. *Journal of clinical and translational science*, *2*(1), 48–52. <https://doi.org/10.1017/cts.2018.9> |
| 101 | Matsas, B., Goralnick, E., Bass, M., Barnett, E., Nagle, B., & Sullivan, E. E. (2022). Leadership Development in U.S. Undergraduate Medical Education: A Scoping Review of Curricular Content and Competency Frameworks. *Academic medicine : journal of the Association of American Medical Colleges*, *97*(6), 899–908. <https://doi.org/10.1097/ACM.0000000000004632> |
| 102 | [Ladhan, Z.](https://www.emerald.com/insight/search?q=Zahra%20Ladhan), [Shah, H.](https://www.emerald.com/insight/search?q=Henal%20Shah), [Wells, R.](https://www.emerald.com/insight/search?q=Ray%20Wells), [Friedman, S.](https://www.emerald.com/insight/search?q=Stacey%20Friedman), [Bezuidenhout, J.](https://www.emerald.com/insight/search?q=Juanita%20Bezuidenhout), [Heerden, B.v.](https://www.emerald.com/insight/search?q=Ben%20van%20Heerden), [Campos, H.](https://www.emerald.com/insight/search?q=Henry%20Campos) and [Morahan, P.S.](https://www.emerald.com/insight/search?q=Page%20S.%20Morahan) (2015), "Global Leadership Model for Health Professions Education – A Case Study of the FAIMER program", [*Journal of Leadership Education*](https://www.emerald.com/insight/publication/issn/1552-9045), Vol. 14 No. 4, pp. 67-91. <https://doi.org/10.12806/V14/I4/R1> |
| 103 | Chan, M. K., Volk, A., Patro, N., Lee, W., Sonnenberg, L. K., Dath, D., & Meschino, D. C. (2022). Creating space for leadership education in undergraduate medical education in Canada. Canadian medical education journal, 13(4), 36–48. <https://doi.org/10.36834/cmej.73216> |
|  | Consultation: we asked to the interviewees or we saw their ORCID. |
| 104 | Berman, P., & Frenk, J. (2018). The New Harvard Doctor of Public Health: Lessons From the Design and Implementation of an Innovative Program in Advanced Professional Leadership. *Public health reports (Washington, D.C. : 1974)*, *133*(6), 759–766. <https://doi.org/10.1177/0033354918804523> |
| 105 | Metcalf, M., Barry, E.S., Mushalko, D., Mushalko, D., Grunberg, N.E. (2023). Innovative leadership & followership in the age of AI: A handbook for creating your future as leader, follower, and AI ally. Portland, OR: Phronesis Publishing (Pty) Ltd. |
| 106 | Barry, E.S., Teunissen, P., & Varpio, L. (2023). Followership in interprofessional healthcare teams: A state-of-the-art review, *BMJLeader*, <https://doi.org/10.1136/leader-2023-000773> |
| 107 | Van Shufflin, M.W., Barry, E.S., Vojta, L., Yarnell, A., & Cole, R. (2023). Students’ perceptions of their leadership development during operation bushmaster. *Military Medicine*. *188* (May/June Supplement), 15-20, <https://doi.org/10.1093/milmed/usac377> |
| 108 | Vojta, L., Roberts, C., Cole, R., Barry, E.S., (2023). Interprofessional experiences in military field practicums: The importance of team integration. *Military Medicine*. 188 (May/June Supplement), 56-62, <https://doi.org/10.1093/milmed/usad106> |
| 109 | Barry, E.S., Durning, SJ, Schreiber-Gregory, D., Grunberg, N.E., Yarnell, A.M, & Dong, T. (2023) Undergraduate medical education leader performance predicts post graduate military leader performance. *Military Medicine*. *188* (May/June Supplement), 87-93, <https://doi.org/10.1093/milmed/usac110> |
| 110 | Grunberg, N.E., McManigle, J.E., Schoomaker, E.B., & Barry, E.S. (2023). Change Leadership in Healthcare, *Clinics in Sports Medicine*. *Clinics in Sports Medicine*. *42*(2), 249-260, <https://doi.org/10.1016/j.csm.2022.11.007> |
| 111 | Barry, E.S., & Grunberg, N.E. (2022). Healthcare teams: Leaders, followers, and teams as a system. In J.F. Quinn, & B.A. White (Eds.), *Cultivating Leadership in Medicine* (2^nd^ ed.). Dubuque, IA: Kendall Hunt Publishing Company. |
| 112 | Barry, E.S., Grunberg, N.E., McManigle, J.E., & Yarnell, A.M. (2022). Uniformed Services University School of Medicine Leader Development Program. In J.F. Quinn, & B.A. White (Eds.), *Cultivating Leadership in Medicine* (2^nd^ ed.). Dubuque, IA: Kendall Hunt Publishing Company. |
| 113 | Grunberg, N.E., & Barry, E.S. (2022). Effective communication in medicine. In J.F. Quinn, & B.A. White (Eds.), *Cultivating Leadership in Medicine* (2^nd^ ed.). Dubuque, IA: Kendall Hunt Publishing Company. |
| 114 | Grunberg, N.E., & Barry, E.S. (2022). From empathy to the aggression-compassion continuum. In S. Ventura (Ed.), *Empathy: Advanced Research and Application*s, InTech. 1-14. |
| 115 | Barry, E.S., Grunberg, N.E., Metcalf, M., Morelli, C., & Morrow-Fox, M. (2022). Innovative Leadership: Leading Post-Pandemic & Beyond. *Amplify*. *35*(6), 6-13. <https://www.cutter.com/article/innovative-leadership-leading-post-pandemic-beyond> |
| 116 | Metcalf, M., Morrow-Fox, M., Morelli, C., Barry, E.S., & Grunberg, N.E. (2022). Innovative leadership makes real impact. *Academia Letters*. Article 5199. <https://doi.org/10.20935/AL5199> |
| 117 | Blickle, J.G., Vojta, L., Huang, C., Wyse, J.M., Yarnell, A.M., & Hartzell, J.D. (2022) Learning to Lead: 10 Leadership Lessons From Operation Bushmaster, *Military Medicine*, usac058, <https://doi.org/10.1093/milmed/usac058> |
| 118 | Barry, E.S., McManigle, J., & McManigle, J.E. (2021). A self-assessment and peer coaching tool for leader development. *Journal of Leadership, Accountability, and Ethics*. 18(4), 1-17. <https://doi.org/10.33423/jlae.v18i4.4605> |
| 119 | Barry, E.S., Larsen, K.B., Meyer, H., Durning, S.J., & Varpio, L. (2021). Leadership and followership in military interprofessional healthcare teams. *Military Medicine*. *186*(S3), 7-15. <https://doi.org/10.1093/milmed/usab118>. |
| 120 | Grunberg, N.E., Barry, E.S., Morrow-Fox, M., & Metcalf, M. (2021). Optimizing Innovative Leadership and Followership. In M. Franco (Ed.), *Leadership: New Insights*, InTech. 1-17. <https://doi.org/10.5772/intechopen.99860>. |
| 121 | Metcalf, M., Barry, E.S., Blakaj, D.M., Fitzpatrick, S., Morrow-Fox, M., & Grunberg, N.E. (2021). *Innovative Leadership for Health Care*. Teaneck, N.J.: Integral Publishers LLC. |
| 122 | Grunberg, N.E. (2021). Life long learning about leadership. *Military Psychologist Newsletter,* March, 2021. |
| 123 | Grunberg, N.E., McManigle, J.E., & Barry, E.S. (2021). Identifying bad, ineffective, and toxic leadership and followership. *Journal of Leadership, Accountability, and Ethics*. 18(1), 69-76, <https://doi.org/10.33423/jlae.v18i1.4004> |
| 124 | Morrow-Fox, M., Barry, E.S., Grunberg, N.E., & Metcalf, M.M. (2021). The innovative health care leader. *Academia Letters*. Article 116, <https://doi.org/10.20935/AL116> |
| 125 | Burchard, M.A., Grunberg, N.E., & Barry, E.S. (2020). Toward understanding and building trust for practicing and emerging healthcare professionals: The ASC-DOC trust model. *MedEdPublish*. *9*(1) 280, <https://doi.org/10.15694/mep.2020.000280.1> |
| 126 | Grunberg, N.E., McManigle, J.E., & Barry, E.S. (2020). Applying social psychology principles to improve healthcare teams. *MedEdPublish*. *9*(1) 290.  <https://doi.org/10.15694/mep.2020.000251.2> |
| 127 | Grunberg, N.E., McManigle, J.E., & Barry, E.S. (2020). Using social psychology principles to develop emotionally intelligent leaders. *Frontiers in Psychology: Personality and Social Psychology*. 11:1917, 1-6. <https://doi.org/10.3389/fpsyg.2020.01917> |
| 128 | Barry, E.S., & Grunberg, N.E. (2020). A conceptual framework to guide leader and follower education, development, and assessment. *Journal of Leadership, Accountability and Ethics*. *17*(1), 127-134. <https://doi.org/10.33423/jlae.v17i1.2795> |
| 129 | Lowe, J.B., Barry, E.S., & Grunberg, N.E. (2020). Improving leader effectiveness across multi-generational workforces. *Journal of Leadership Studies*. *14*(1), 46-52. <https://doi.org/10.1002/jls.21681> |
| 130 | Tayne, S., Hutchinson, M.R., O’Connor, F.G., Taylor, D.C., Musahl, V., & Indelicato, P. (2020) Leadership for the team physician. *Current Sports Medicine Reports*. 19(3), 119-123. <https://doi.org/10.1249/JSR.0000000000000696> |
| 131 | O'Connor, F.G., Grunberg, N.E., Harp, J.E., & Deuster, P.A. (2020). Exertion-related illness: The critical roles of leadership and followership. *Current Sports Medicine Reports*. *19*(1), 35-39. <https://doi.org/10.1249/JSR.0000000000000673> |
| 132 | Barry, E.S., Hudepohl, N., Kleber, H.G., McManigle, J.E., Weistroffer, J.K., & Grunberg, N.E. (2019). Importance of integration of leader and leadership education and development in medical education across the life-cycle. *MedEdPublish*. 8, [3], 69.  <https://doi.org/10.15694/mep.2019.000218.1> |
| 133 | Barry, E.S., & Grunberg, N.E. (2019). Healthcare teams. In J.F. Quinn, & B.A. White (Eds.), *Cultivating Leadership in Medicine*. Dubuque, IA: Kendall Hunt Publishing Company, 117-130. |
| 134 | Barry, E.S., & Grunberg, N.E. (2019). Healthcare teams. In J.F. Quinn, & B.A. White (Eds.), *Cultivating Leadership in Medicine*. Dubuque, IA: Kendall Hunt Publishing Company, 117-130. |
| 135 | Barry, E.S., & Grunberg, N.E., & McManigle, J.E. (2019). Uniformed Services University School of medicine leader and leadership education and development. In J.F. Quinn, & B.A. White (Eds.), *Cultivating Leadership in Medicine*. Dubuque, IA: Kendall Hunt Publishing Company, 141-154. |
| 136 | Grunberg, N.E., & Barry, E.S. (2019). Effective communication. In J.F. Quinn, & B.A. White (Eds.), *Cultivating Leadership in Medicine*. Dubuque, IA: Kendall Hunt Publishing Company, 77-89. |
| 137 | Callahan, C., & Grunberg, N.E. (2019). Military medical leadership.  In: F.G. O'Connor, E.B. Schoomaker, D.C. Smith (Eds.), *Fundamentals of Military Medicine*. San Antonio, TX: Borden Institute, 51-66. |
| 138 | Yarnell, A.M., Dullea, C., & Grunberg, N.E. (2019). Military communication.  In: F.G. O'Connor, E.B. Schoomaker, D.C. Smith (Eds.), *Fundamentals of Military Medicine*. San Antonio, TX: Borden Institute, 165-178 |
| 139 | Barry, E.S., Grunberg, N.E., & Kleber, H.G. (2018). Approaches for curriculum and assessment in leader and leadership education and development programs in American medical schools. *MedEdPublish*, 7, [4], 23. <https://doi.org/10.15694/mep.2018.0000244.1> |
| 140 | Grunberg, N.E., Barry, E.S., Callahan, C.W., Kleber, H.G., McManigle, J.E., & Schoomaker, E.B. (2018). A conceptual framework for leader and leadership education and development. *International Journal of Leadership in Education*, *22*(5), 1-7. <https://doi.org/10.1080/13603124.2018.1492026> |
| 141 | Grunberg, N.E., Barry, E.S., Kleber, H., McManigle, J.E., & Schoomaker, E.B., (2018). Charting a course for leader and leadership education and development in American medical schools. *MedEdPublish, 7 (1) 37*. <https://doi.org/10.15694/mep.2018.0000037.1> |
| 142 | Yarnell, A.M., & Grunberg, N.E. (2017). Developing "Allostatic Leaders": A Psychobiological Perspective. In M. Clark & C.W. Gruber (Eds.), Leader Development Deconstructed. Cham, Switzerland: Springer International Publishing, 23-50 |
| 143 | Grunberg, N.E., Barry, E.S., Kleber, H., McManigle, J.E., & Schoomaker, E.B., (2017). Seven steps to establish a leader and leadership education and development (LEAD) program. In M. Clark & C.W. Gruber (Eds.), Leader Development Deconstructed. Cham, Switzerland: Springer International Publishing, 301-321. |
| 144 | Eklund, K.E., Barry, E.S., & Grunberg, N.E. (2017). Gender and Leadership. In A. Alvinius (Ed.), Gender Differences in Different Contexts, InTech, 129-150 |
| 145 | O’Connor, F., Grunberg, N.E., Kellermann, A., & Schoomaker, E. (2015).  The USUHS school of medicine leadership education and development program (LEAD).  Military Medicine, *180*(4), 147-152. <https://doi.org/10.7205/MILMED-D-14-00563> |
| 146 | Pottie, K., & Gruner, D. (2023). Community-based care for refugees to overcome health disparities. *Nature reviews. Disease primers*, *9*(1), 16. https://doi.org/10.1038/s41572-023-00430-9 |
| 147 | Stoller J. K. (2021). Leadership Essentials for CHEST Medicine Professionals: Models, Attributes, and Styles. *Chest*, *159*(3), 1147–1154. <https://doi.org/10.1016/j.chest.2020.09.095> |
| 148 | Stoller JK. (2021) Developing physician leaders: Why, whether, and is it effective? *Can Phys Leader J* , 7(2), 85-88. https//[doi.org/10.397964/cr24734](https://l.facebook.com/l.php?u=http%3A%2F%2Fdoi.org%2F10.397964%2Fcr24734%3Ffbclid%3DIwAR2oktWUBgpsnZbQG5eBaAJjQnpZ51tHh1h40TxtBLJWNgLrggTKeaK4WvI&h=AT18GzlTSlMDFWo1g87FF3XtxQrubnAJyy2SWYnQTR00uRiW-1FM-5m0MJayOCzY4C2W19eoH2FVq_k6cwlBa_jLu4SRVVTCu6yKtlRiTF5ZAdEahbmTr1oNNwjAPqjxCDs) |
| 149 | Stoller, J. K., Taylor, C. A., & Farver, C. F. (2013). Emotional intelligence competencies provide a developmental curriculum for medical training. *Medical teacher*, *35*(3), 243–247. <https://doi.org/10.3109/0142159X.2012.737964> |
| 150 | Abdel-Razig, S., & Stoller, J. K. (2023). Global "systemness" in medical education: A rationale and framework to assess performance. *Medical teacher*, 1–5. Advance online publication. <https://doi.org/10.1080/0142159X.2023.2244665> |
| 151 | Accreditation Council for Graduate Medical Education.Milestones Guidebook for Residents and Fellow. 2020 . Available from: [MilestonesGuidebookforResidentsFellows.pdf (acgme.org)](https://www.acgme.org/globalassets/PDFs/Milestones/MilestonesGuidebookforResidentsFellows.pdf?fbclid=IwAR2UpdZzSOyDom2UDpmctZBQenl6pwe56Bu_W8-EEWQ2F0tZwVrJFdE0_wo) Accessed on December 6th 2023 |
| 152 | Bertelsen, N. S., DallaPiazza, M., Hopkins, M. A., & Ogedegbe, G. (2015). Teaching global health with simulations and case discussions in a medical student selective. *Globalization and health*, *11*, 28. <https://doi.org/10.1186/s12992-015-0111-2> |
| 153 | Spencer J, McNulty A, Brice A. (2010). Providing Care for ‘hard to reach out to’ Patient Groups. *Medical Education*,44(3):52. |
| 154 | Organizacion Panamericana de la Salud. Aportes y desafíos en el proceso de reforma del sector salud con énfasis en territorios prioritarios Colombia 2022- Available from: <https://www.saludpublicacolombia.org/wp-content/uploads/2023/05/Aportes-y-desafios.pdf?fbclid=IwAR2HQ_I9jGKP0LTxU_VcBTNW6p5FJqs72D-LRUgwY-SgzCTb8bMJwJnwfYw> Accessed on December 6th 2023 |
| 155 | Lamus-Lemus, F., Correal-Muñoz, C., Hernandez-Rincon, E., Serrano-Espinosa, N., Jaimes-deTriviño, C., Diaz-Quijano, D., & García-Manrique, J. G. (2017). The pursuit of healthier communities through a community health medical education program. *Education for health (Abingdon, England)*, *30*(2), 116–125. <https://doi.org/10.4103/efh.EfH_283_14> |
| 156 | Hernández-Rincón EH, Lamus-Lemus F, Carratalá-Munuera C, et al. (2017). Building community capacity in leadership for primary health care in Colombia. *MEDICC Review*,19(2-3),65-70. [mrw172_3k.pdf (medigraphic.com)](https://www.medigraphic.com/pdfs/medicreview/mrw-2017/mrw172_3k.pdf) |
| 157 | Soriano-Moreno, Anderson N., Romero-Robles, Milton A., Perez-Fernandez, Jhosuny, Muñoz del Carpio-Toia, Agueda, & Toro-Huamanchumo, Carlos J.. (2021). Estudiantes de medicina como impulsores de la educación médica: el caso de la Sociedad Científica Médico Estudiantil Peruana. *Revista Habanera de Ciencias Médicas*, *20*(1), . Epub 10 de marzo de 2021. Recuperado en 21 de agosto de 2024, de <http://scielo.sld.cu/scielo.php?script=sci_arttext&pid=S1729-519X2021000100013&lng=es&tlng=es>. |

**Figure 1. PRISMA diagram: databases, registries, handsearching, and consultation.**

**Identification of studies via other methods**

**Identification of studies via databases and registers**

Records identified from:

Consultation and ORCID(n = 54)

Records removed *before screening*:

Duplicate records removed (n = 216 )

Records identified from: 4765

Medline (n = 4520)

Embase (n = 75)

LILACS (n = 70)

Google (n = 100)

**Identification**

Records screened

Title (n = 4549)

Records excluded by title

(n = 4224)

Record excluded by title, abstract and duplicated records (n= 0)

Record screened by title, abstract (n = 54)

Records screened

Abstract (n =325)

Records excluded by abstract (n = 163)

**Screening**

Reports excluded:

219 No leadership education in undergraduate medical education

2 missing

1 published ≤2009

Records screened full text (n =325)

Reports assessed for eligibility.

(not applicable)

Databases: (n= 103)

Consultation: (n = 54)

Total: (n = 157)

**Included**
