# Supplementary material for: The Meaning of Leadership in Medical Education in the Pan American Health Organization Member States: A Stakeholder Analysis and Interviews
Source: Int J Public Health. 2026 Feb 26;71:1608502. doi: 10.3389/ijph.2026.1608502 (PMC12979235; doi:10.3389/ijph.2026.1608502)
Supplement: Supplementary file 5 [file Supplementaryfile7.docx]

**Supplementary material 7.** Authors reflection on our own positionality about leadership development.

Table. Authors´ disciplinary backgrounds

| Author | Disciplinary backgrounds |
| --- | --- |
| PRF | He was PhD candidate in the Department of International Health in the Netherlands. His PhD focuses on leadership competency development in undergraduate medical education. His research examines leadership competency-based education in diverse workforces, including health, public health, and allied health professions. He has a special interest in interdisciplinary and transprofessional education, conducting reviews, and promoting knowledge translation. He works at a university in Colombia and a university in the Netherlands in undergraduate medical education and a Master of Public Health programme. He serves as a peer reviewer for the Pan American Journal of Public Health and for Public Health Reviews. |
| NGN | An undergraduate Psychology student at a university in Colombia. In addition to her major, she has pursued minors in Literature and Creative Writing. She has collaborated alongside PRF and a university in the Netherlands, researching leadership competency-based education in Undergraduate Medical Education (UME) with an inter- and trans-professional approach. She has also peer-reviewed a manuscript submitted to the Pan American Journal of Public Health. Currently, she is applying for a master’s in public policy in Germany. |
| SGA | An undergraduate Psychology student in Colombia. In addition to her major, she has pursued minors in Neuroscience and Gender and Sexuality. She actively engages in diverse research groups, contributing to literature reviews and the development of psychosocial interventions. She has collaborated with PRF and a university in the Netherlands on research in leadership competency-based education in Undergraduate Medical Education (UME) with an inter- and trans-professional approach. |
| MP | A lecturer and PhD researcher in the Department of International Health at a university in the Netherlands. She holds Master of Science degrees in Social and Cultural Anthropology and in Governance and Leadership in European Public Health from universities in the Netherlands. |
| SB | Associate Dean of Global Health and Professor of Community and Global Health at a university in the USA. She directs a professional distance doctoral program in Global Health Leadership for mid- to senior-level health practitioners worldwide. She holds faculty positions at universities in the Netherlands and France and serves as Chair of the Board of Accreditation for the European Agency for Public Health Education Accreditation (APHEA). She holds a doctorate in public health from a university in the USA. |
| LMV | An academic leader committed to higher education in international settings. She has focused on the transformation and innovation of educational organizations. Her interpersonal skills have enabled her to strengthen organizations by leveraging individual and collective capacities, grounded in values and ethics. She has served as president and CEO of a public health association, leading organizational changes during multiple public health crises, forming global partnerships, and advancing the work and voice of academic public health. She has also directed schools of public health in Mexico and implemented major educational projects for UNICEF in Central America, developing innovative academic programs and services. |
| LJHF | An associate professor at a school of medicine in Colombia. He has worked in the Colombian government, multiple universities, and the Pan American Health Organization. He directs a public health department at a university in Colombia and teaches in undergraduate and postgraduate programs. His research focuses on knowledge translation, environmental health, education, bioethics, and healthcare systems. He is a member of international and national public health associations. |
| KC | A professor of Public Health Leadership and Workforce Development and head of a department at a university in the Netherlands. She is an Honorary Member of the UK Faculty of Public Health and a past president of a European public health schools association. She is a lead author of a WHO-ASPHER Competency Framework and the Road Map to Professionalising the Public Health Workforce in the European Region. |

Box. Positionality about leadership development.

| - One cannot educate or train someone in what has not been previously conceptualized. A qualitative exercise is needed to survey the perceptions of key stakeholders in order to conceptualize leadership in the Pan American Health Organization member states, with a focus on Latin America and the Caribbean. - Key stakeholders working in the United States and Canada should also be included in the conceptualization, as they have experience in the field, share borders with Latin American and Caribbean countries, and face joint challenges in improving the health and well-being of people. - The conceptualization of leadership should include the target audiences to be educated and trained, as well as the purpose of leadership—that is, the challenges that can be addressed through leadership development. - Leadership education and training must break down professional silos. - Leadership education and training should be accessible to everyone, including individuals studying to become professionals, those who are already professionals, and those who have not had the privilege or opportunity to attend university. - A second stage of the leadership development process would involve proposing a competency-based framework aligned with the conceptualization of leadership for PAHO member states. |
| --- |
