# Supplementary material for: The Meaning of Leadership in Medical Education in the Pan American Health Organization Member States: A Stakeholder Analysis and Interviews
Source: Int J Public Health. 2026 Feb 26;71:1608502. doi: 10.3389/ijph.2026.1608502 (PMC12979235; doi:10.3389/ijph.2026.1608502)
Supplement: Supplementary file 7 [file Supplementaryfile4.docx]

**Supplementary material 4. Stakeholders’** **Profile**

**Table 1. Stakeholders´ characteristics**

| # | Sampling | Gender  Masculine (M)  Feminine (F) | Internal(I) Andes, Indiana o Maastricht.  External (E)  Another organization | Language  Spanish(S) English (E) | In person (P)  Online(O) |
| --- | --- | --- | --- | --- | --- |
| 1 | S3 | M | E | S | O |
| 2 | S1 | F | E/I | S | O |
| 3 | S3 | F | E/I | S | O |
| 4 | S1 | M | E | S | O |
| 5 | S1 | M | E | S | O |
| 6 | S3 | F | I | S | O |
| 7 | S2 | M | E | S | O |
| 8 | S1 | F | E | S | O |
| 9 | S2 | F | E | S | O |
| 10 | S1 | M | E | S | O |
| 11 | S1 | F | I | S | P |
| 12 | S1 | M | E | S | O |
| 13 | S2 | F | E | S | O |
| 14 | S1 | M | E | S | O |
| 15 | S1 | F | E | S | O |
| 16 | S2 | M | E | S | P |
| 17 | S1 | M | E | S | O |
| 18 | S3 | M | E | S | O |
| 19 | S2 | F | E | S | O |
| 20 | S1 | M | E | E | O |
| 21 | S1 | F | E | E | O |
| 22 | S1 | F | E | E | O |
| 23 | S2 | M | E | S | O |
| 24 | S1 | F | E | E | O |
| 25 | S1 | F | E | E | O |
| 26 | S1 | M | E | E | O |
| 27 | S1 | M | E | E | O |
| 28 | S1 | M | E | E | O |
| 29 | S1 | M | E | S | O |
| 30 | S1 | M | E | E | O |
| 31 | S1 | F | E | E | O |
| 32 | S1 | F | E | E | O |
| 33 | S1 | F | E | E | O |
| 34 | S1 | F | E | S | O |
| Total:34 | S1: 25(74%)  S2: 6 (18%)  S3: 3 (9%) | F: 17(50%)  M: 17(50%) | I: 2 (6%)  E: 29 (85%)  E and I: 2 (6%) | S: 22 (65%)  E: 12(35%) | P: 2 (6%)  Online: 32 (94%) |

Note: the stakeholder number is not the same in tables 2, 3, and 4.

**Table 2. Stakeholders´ location**

| # | Location |
| --- | --- |
| 1 | US |
| 2 | Canada |
| 3 | Mexico |
| 4 | Chile |
| 5 | Colombia |
| 6 | Mexico |
| 7 | Colombia |
| 8 | Colombia |
| 9 | Mexico |
| 10 | Mexico |
| 11 | Mexico |
| 12 | Mexico |
| 13 | Colombia |
| 14 | Peru |
| 15 | Cuba |
| 16 | Colombia |
| 17 | Colombia |
| 18 | Bolivia |
| 19 | Peru |
| 20 | Peru |
| 21 | US |
| 22 | US |
| 23 | US |
| 24 | US |
| 25 | US |
| 26 | US |
| 27 | Canada |
| 28 | Peru |
| 29 | US |
| 30 | Mexico |
| 31 | Canada |
| 32 | US |
| 33 | US |
| 34 | Canada |
| Total:34 | US: 10 (29%)  Mexico: 7 (21%)  Colombia: 6 (18%)  Peru: 4 (12%)  Canada: 4(12%)  Chile: 1 (3%)  Cuba: 1 (3%)  Bolivia: 1 (3%) |

Note 1: Do not confuse this with nationality, since that was not asked; the country refers to where the stakeholder is affiliated.

Note 2: The stakeholder number is not the same in tables 1, 3, and 4.

**Table 3. Stakeholders’ affiliations**

| # | National | | | Multilateral with legal bounds in members states in the Americas. | |
| --- | --- | --- | --- | --- | --- |
|  | Universities | Hospitals, clinics & medical centers | Associations, academies, Societies & Foundations | Pan American Health Organization | Andean Health Body-Hipólito Unanue Convention |
| 1 | X |  |  |  |  |
| 2 | X |  |  |  |  |
| 3 | X | X |  |  |  |
| 4 |  | X |  |  |  |
| 5 | X |  | X | X |  |
| 6 | X |  |  |  |  |
| 7 | X |  |  |  |  |
| 8 | X |  |  |  |  |
| 9 | X |  |  |  |  |
| 10 | X | X | X |  |  |
| 11 | X |  |  |  |  |
| 12 | X |  |  |  |  |
| 13 | X |  |  |  |  |
| 14 | X |  |  |  |  |
| 15 | X |  | X |  |  |
| 16 |  |  | X |  |  |
| 17 | X |  |  |  |  |
| 18 | X |  |  |  |  |
| 19 | X |  | X |  |  |
| 20 | X | X |  |  |  |
| 21 | X |  |  |  |  |
| 22 | X | X |  |  |  |
| 23 |  | X |  |  |  |
| 24 | X | X |  |  |  |
| 25 | X |  |  |  |  |
| 26 | X |  |  |  |  |
| 27 | X | X |  |  |  |
| 28 | X |  | X |  |  |
| 29 | X |  | X |  |  |
| 30 | X |  |  |  |  |
| 31 | X |  |  |  |  |
| 32 | X |  |  |  |  |
| 33 | X |  |  |  |  |
| 34 |  |  |  |  | X |
| Total | 30/34, 82% | 8/34, 24% | 7/34, 21% | 1/34, 3% | 1/34, 3% |

Note: The number of the box is not related to the code of the interview. The number of appearances is random.

Note: the stakeholder number is not the same in tables 1, 2, and 4.

**University appointments**:

- Professor of Physiology / Faculty of Medicine
- Workshop Professor / Faculty of Sciences
- Collaborator at the Center for Complexity Sciences
- Research Coordinator of University Engagement for Health
- Undergraduate teaching: Microbiology for Dentistry
- Lecturer in Microbiology and Pharmacology for undergraduate and postgraduate Dentistry
- Lecturer in Periodontics specialization, School of Dentistry
- Representative of the School of Dentistry at the Center for Medical Education and Health Sciences
- Teacher in the Master’s in Medical Education and Health Sciences program (courses in Assessment and Educational Research)
- Lecturer, Department of Family Medicine and Public Health / Faculty of Medicine
- Academic, Department of Physiology / Faculty of Medicine
- Coordinator of Educational Research, Subdivision of Medical Specializations / Faculty of Medicine
- Professor, Department of Physiology and Department of Psychiatry and Mental Health / Faculty of Medicine
- Behavioral Neurophysiology Laboratory
- Head, State Department of Teaching and Research
- Vice Dean of Research / Faculty of Social Sciences
- Professor, Department of Psychology / Faculty
- Lecturer-Researcher, School of Medicine
- Researcher at the Latin American Center of Excellence in Climate Change and Health, Faculty of Public Health and Administration
- Professor, Graduate Division, Faculty of Medicine
- Professor, Department of Public Health / School of Medicine
- Professor of Family Medicine and Epidemiology and Biostatistics
- Chair in Family Medicine Studies
- Professor in the Department of Family Medicine
- Professor in Family Medicine, Epidemiology and Community Medicine
- Dean of a Faculty of Medicine
- Lecturer in Medical Physiology
- Lecturer in Health Administration
- Medical student
- Professor of Psychiatry, Department of Psychiatry and Behavioral Sciences
- Professor of Physiology, Department of Physiology / School of Medicine
- Director of Education and Faculty Development (teaching, administration & educational scholarship in medical & health professions education)
- Center for Health Professions Education
- Professor in the Department of Anesthesiology
- Faculty mentoring
- Leadership Coach
- Professor of Organizational Behavior
- President of a university
- Professor of Public Health Sciences / School of Medicine
- Professor of Health Sector Management and Policy / Business School
- Professor of Sociology / College of Arts and Sciences
- Professor of Health Studies / School of Nursing and Health Studies
- Professor in the Graduate School of Nursing
- Professor in the Department of Emergency Medicine
- Chair, Department of Emergency Medicine
- Professor, Section of Medical Science
- Professor of Medical & Clinical Psychology / School of Medicine
- Professor of Neuroscience / School of Medicine
- Medical School leadership team for faculty and faculty development
- Medical Education Fellowship Director, Department of Emergency Medicine
- Professor of Healthcare Management / School of Business
- Lecturer in the Department of Global Health and Social Medicine
- Executive Dean for Education and Policy at a university
- Department of Medical Education
- Professor of Medical Education and Pediatrics
- Professor of Military and Emergency Medicine / School of Medicine
- Professor at a university / School of Nursing
- Director of Research and Development, Leadership Education and Development program
- Working at the Office of Leadership Education / Faculty of Health Sciences
- Director of Faculty Development, Department of Military and Emergency Medicine; Chair, Faculty Mentoring and Development, Department of Anesthesiology; and Consultant for Faculty Promotions, Department of Surgery

**Hospitals, clinics, and medical centers appointments:**

- Coordinator of Teaching and Research at a hospital
- Clinical Professor in Internal Medicine
- Long-term psychotherapy groups for patients with chronic illnesses
- Pediatric Hospitalist / Faculty of Medicine & Dentistry / School of Medicine
- Professor of Family Medicine
- Chair of the Education Institute in a hospital (covering Continuing Medical Education, the Clinic Lerner College of Medicine, Scientific Publications, and the Center for Educational Resources)
- Samson Global Leadership Academy Endowed Chair
- Pulmonary/Critical Care Physician
- Attending Physician, Emergency Medicine

**Associations/Academia/societies appointments** :

- Member of the Colombian Association of Public Health
- Member of the National Academy of Medical Education
- Member of the State of Mexico Academy of Medicine
- Member of the Mexican Society of Physiological Sciences
- Member of the Board of Directors, Colombian Association of Epidemiology (ASOCEPIC)
- Member of the Colombian Association of Faculties of Medicine
- Member of the Peruvian Scientific Medical Student Society (SOCIMEP)
- Member of the Academy of Medicine of Washington, D.C.
- Member of the Association for Psychological Science
- Member of the American Psychological Association
- Member of the Academy of Behavioral Medicine Research
- Member of the Society of Behavioral Medicine
- Member of the American Academy of Arts and Sciences
- Member of the U.S. National Academy of Medicine
- Member of the National Academy of Medicine of Mexico
- Member of El Colegio Nacional
- Member of the United Nations Foundation
- Member of the Robert Wood Johnson Foundation
- Member of the Institute for Health Metrics and Evaluation

**PAHO:**

- International Organization of Telemedicine and Telehealth, and the Virtual Campus of Public Health of the Pan American Health Organization (PAHO/WHO) in Colombia

**ORAS CONHU** :

- Head of Continuing Education and the Andean Committee on Human Resources in Health at the Andean Health Organization (ORAS-CONHU)

**Table 4. Stakeholder´s academic background**

|  |  |  | Postgraduate education | | | |
| --- | --- | --- | --- | --- | --- | --- |
|  | Graduate education | Undergraduate education | Master | DrPH/PHD | Residency | Fellowship |
| 1 |  | X |  | X |  |  |
| 2 |  | X | X | X |  |  |
| 3 |  | X | X |  | X |  |
| 4 |  | X | X |  | X | X |
| 5 |  | X | X | X |  |  |
| 6 |  | X | X |  |  |  |
| 7 |  | X | X | X |  |  |
| 8 |  |  |  |  |  |  |
| 9 |  | X | X | X | X |  |
| 10 |  | X | X | X |  |  |
| 11 |  | X |  |  |  |  |
| 12 |  | X | X |  |  |  |
| 13 |  |  |  | X |  |  |
| 14 | X |  |  |  |  |  |
| 15 |  | X | X | X |  |  |
| 16 |  | X | X |  |  |  |
| 17 |  | X | X |  | X |  |
| 18 |  | X | X |  |  |  |
| 19 | X |  |  |  |  |  |
| 20 |  | X | X |  | X | X |
| 21 |  | X |  | X |  |  |
| 22 |  | X | X |  | X |  |
| 23 |  | X |  |  |  |  |
| 24 |  | X | X |  | X | X |
| 25 |  | X | X | X |  |  |
| 26 |  | X |  |  | X |  |
| 27 |  | X | X |  | X | X |
| 28 |  | X | X | X |  |  |
| 29 |  | X | X | X |  |  |
| 30 |  | X |  |  | X |  |
| 31 |  | X | X |  |  |  |
| 32 |  | X |  | X |  |  |
| 33 |  | X | X |  | X |  |
| 34 |  | X | X |  |  |  |

Note: The number of the box is not related to the code of the interview. The number of appearances is random.

**Graduate education:**

- Medical student

**Undergraduate education:**

- Undergraduate medical education.
- General dentistry
- Bachelor on Pharmacy degree.
- Bachelor of Artes degree in Psychology
- Bachelor’s degree in public administration
- Biology
- Biomedical Engineering
- Medical Microbiology
- Bachelor of Arts
- Bachelor of Artes degree in Social Communication

**Postgraduate education: Master**

- Master’s in business administration; and Executive MBA.
- Master’s in medical education; Master's Degree in Technological Education and Technopedagogy; Masters’ degree in education; Master’s in/of Health Professions Education; Master´s of Education/ Med- health professions and related clinical science; and Master's in Higher Education in Health.
- Master´s of Science (M.Sc.) Medical Anthropology.
- Master´s of Public Health.
- Master´s in clinical science.
- Master of Science Biomedical engineering.
- Master’s degree in organizational development and analysis.
- Master in Big Data and Business Intelligence.
- MSc in epidemiology and Master of Science in Epidemiological and Clinical Research
- M.S. in Physiological Science.
- M.S. in Pharmacology.
- MSc Primary Care Research.
- M.S. in Medicine, Dentistry and Health Sciences.
- Master's in Governance and Management of the Healthcare System
- Master's in Analysis of Contemporary Political, Economic, and International Problems

**Postgraduate education: PhD**

- PhD Epidemiology.
- Ph.D. in Physiology.
- Ph.D. Health Professions Education.
- Ph.D. in Medical Care Organization and in Sociology.
- Ph.D. Business/ management.
- Ph.D. in Biomedical Sciences.
- Ph.D. in Clinical Research.
- Ph.D. in Pharmacological Sciences.
- Doctor of Philosophy (Medicine, Dentistry and Health Sciences).
- PhD in Social Sciences.
- PhD in Clinical and Health Psychology.
- Doctorate in Leadership and Management of Higher Education Institutions.

**Postgraduate education: Residency**

- Internal medicine.
- Family medicine.
- Psychiatry.
- Pediatrics.
- Emergency Medicine.
- Periodontics.

**Fellowship:**

- Infectious Disease.
- Psychiatric genetics.
- Pulmonary/critical care medicine.
- Critical care medicine/anesthesiology.
- Medical education fellowship.

**Table 5. Stakeholders’ expertise.**

| # | Surname and names. | Affiliation(s) | Expertise in the field. |
| --- | --- | --- | --- |
| 1 | Azzam, Amin | 1. University of California, San Francisco 2. University of California, Berkeley 3. Samuel Merritt University | 1. School of Medicine, Department of Psychiatry & Behavioral Sciences 2. School of Public Health, UC Berkeley – UCSF Joint Medical Program 3. Health Sciences Simulation Center |
| 2 | Barry, Erin | Uniformed Services University, Bethesda, MD, USA | Erin S. Barry is Assistant Professor in the Department of Anesthesiology at the Uniformed Services University (USU). She has a secondary appointment as Assistant Professor in the USU Department of Military and Emergency Medicine as well as the Center for Health Professions Education. She is a health professions education researcher who develops and delivers curriculum and education assessments, and conducts research and scholarship related to leadership, followership, and healthcare teams She has contributed to leadership education, development, assessment, scholarship, and online learning at USU since 2014. Additionally, she is a Leadership Coach. |
| 3 | Chandran, Latha | University of Miami Miller School of Medicine | Medical Education, Faculty Development, Educational Scholarship |
| 4 | Frenk, Julio | University of Miami | President; Co-Chair of the Lancet Commission on the Education of Health Professionals for the 21^st^ Century (1^st^ author of its report) |
| 5 | Grunberg Neil E | - Professor of Military and Emergency Medicine and Professor of Neuroscience in the Uniformed Services University (USU) School of Medicine - Professor in the USU Graduate School of Nursing - Director of Research and Development in the USU Leadership Education and Development (LEAD) program, Bethesda, Maryland - Director of Faculty Development for the Department of Military and Emergency Medicine; as Chair, Faculty Mentoring and Development, Department of Anesthesiology   as Consultant for Faculty Promotions, Department of Surgery | He has been educating physicians, psychologists, and nurses for the Armed Forces and Public Health Service and scientists for research and academic positions since 1979.  He is a co-founder of the Healthcare Leadership Community of the International Leadership Association, a co-founder of the World Health Leadership Network, and a member of Teaching Followers Courage.  He is a co-author of *Innovative Leadership for Health Care* (2021) and *Innovative Leadership and Followership in the Age of AI: A Guide to Creating Your Future as Leader, Follower, ad AI Ally.* (2023). |
| 6 | Isbej Espósito, Lorena | 1. School of Dentistry, Faculty of Medicine, Pontificia Universidad Católica de Chile. 2. School of Health Professions Education, Maastricht University, The Netherlands | Representative of the School of Dentistry at the Center for Medical Education and Health Sciences.  Expert in Faculty Development for medical educators.  Teacher of the Master in Medical Education and Health Sciences in courses of Assessment and educational research.  Teacher of the Diploma in Medical Education and Health Sciences in Curriculum Development course. |
| 7 | Lema-Vélez, Mariana. | Clinical teacher. School of Medicine. University of the Andes (Bogotá, Colombia) | My expertise on leadership and its related competencies lies in the leadership and communication domain, particularly in effective written and oral communication and presentation skills. Furthermore, I introduce medical students to the role of non-verbal communication and to communication with diverse cultures (Intercultural competence) and disciplines.  I believe that undergraduate and postgraduate medical students should receive effective and goal-oriented training in all leadership domains and from different perspectives such as social, humanist, political, and clinical ones. |
| 8 | Romero Robles, Milton A. | Scientific University of the South, Faculty of Health Sciences, Medicine. Lima, Perú | Medical Surgeon and RENACYT VI Researcher. Trained in the evaluation of systematic AMSTAR-II reviews and AGREE-II clinical practice guidelines by the Health Technology Assessment and Research Institute (IETSI - EsSalud). Methodologist and author of two clinical practice guidelines developed using the GRADE methodology. Recipient of the scholarship awarded by the Evidence Foundation (USA) for the Systematic Review Workshop organized by the GRADE community in the United States. Current Cochrane member and translation coordinator for the Students for Best Evidence (ExME) initiative supported by Cochrane Iberoamérica |
| 9 | Pottie, Kevin | Dalhouise University, Halifax,Nova Scotia Canada | Professor and Research Chair of Family Medicine, Dalhousie University, Halifax, NS Canada. Global and refugee health education: [www.ccirhken.ca](http://www.ccirhken.ca/) |
| 10 | Salas Gonzalez, Yadira Josefina | Andean Health Body-Hipólito Unanue Convention. | Professional Experience:   - Head of Continuing Education and Coordinator of the Andean Committee on Human Resources in Health at the Andean Health Organization (ORAS-CONHU). - Tutor for the Impact Leadership Course on Leadership for Policy Management, Regulation, and Planning in the Andean Region. - Virtual tutor, facilitator, coordinator, and instructional designer for various health training courses and programs, both in-person and virtual, at the PAHO Virtual Campus (Regional Node and Venezuela), University of Health Sciences Venezuela, University of Lanus Argentina, University of Buenos Aires Argentina, and National University of San Marcos. - Author of the Continuing Education in Health component of the Andean Health Human Resources Policy and Plan of the Andean Health Organization – Hipólito Unanue Agreement. - Author and compiler of publications on Human Resources in Health: “Changes in Health Science Curricula During the Pandemic,” “Conditions Influencing the Job Choices of Health Personnel,” among others. - Expertise in knowledge management in health and in technopedagogy processes supporting graduate programs in Public Health Management, Occupational Health and Workplace Environmental Hygiene, Comprehensive Adolescent Health, Epidemiology, and Comprehensive General Medicine   **Academic Background:**  Master's in Technopedagogy and Virtual Education (Mexico). Master's in Big Data and Business Intelligence (Spain). International MBA, Master of Business Administration (USA). Postgraduate Diploma in Education, Media, Information and Communication Technology (Argentina). Bachelor's Degree in Communication for Social Development (Venezuela. Expert in Technopedagogy, E-learning, and Digital Media (Ecuador) with Diplomas in Occupational Health; Gender, Health, and Citizenship Building; Educational Research with ICT; Social Communication and Collective Health; and Global Health. |
| 11 | Sampieri Cabrera, Raúl | Learning Sciences Laboratory, Department of Physiology, Faculty of Medicine, UNAM. | I am currently head researcher of the Learning Sciences laboratory at the Faculty of Medicine, UNAM. In the laboratory, I lead a team comprised of 1 research technician, 2 postdoctoral students, 3 master's students in medical education, 1 doctoral student, 7 undergraduate students in a medical research program, and 1 laboratory assistant. In addition, I coordinate 2 international diploma programs related to learning sciences, mental health, and medical education. I am a clinical research leader in the health community of the University Corporation for Internet Development where I coordinate research teams to consolidate protocols and projects across various national and international universities.  I serve as an associate editor for the Directory of Open Access Journals (DOAJ), where I ensure that the journals applying meet the editorial quality to be included in the index.  I have published 4 books related to medical education in the areas of physiology and mental health, and I served as a guest editor for a special issue of the Mental Health journal of Mexico on a monothematic related to mental health and health sciences education in Latin America.  I am the Clinical Research Coordinator at the Angeles Lindavista Hospital in Mexico.  I also participate as an associate researcher in the Conductome project at the Center for Complexity Sciences at UNAM.  Currently, I hold the Special Dr. Aniceto Orantes Chair granted by the Commission for University Merit of the Honorable Technical Council of the Faculty of Medicine of UNAM, for the project "Promotion of socio-emotional skills for academic and mental well-being in medical students". |
| 11 | Sonnenberg, Lyn K. | Professor Emerita at the University of Alberta. University of Alberta. | Her leadership experience includes being Vice President of the Medical Council of Canada (MCC), National Director, Learning Transformation at the Royal College of Physicians and Surgeons of Canada, Associate Dean, Educational Innovation & Academic Technologies at the University of Alberta, and Medical Director of Infant and Preschool Services for the Glenrose Rehabilitation Hospital in Alberta, Canada. She is currently the Director, Learning Strategies, for the Equity in Health Systems (EqHS) Lab in Ottawa, Canada. |
| 12 | Stoller, James K. Stoller, MD, MS  Professor and Chairman, Education Institute | Cleveland Clinic | Leadership Development, Pulmonary Medicine |
| 13 | Torruco García, Uri | Infectologist of the HIV program in the state of Veracruz in Mexico. | Political leadership, Collaborative leadership, Leadership and communication, leading change, Emotional intelligence and leadership in team-based organizations, Leadership, organizational learning and development |
| 14 | Vieyra-Reyes Patricia | Neurophysiology of Behavior Laboratory, Center for Research and Advanced Studies in Animal Health, Faculty of Veterinary Medicine and Zootechnics, Autonomous University of the State of Mexico. | I am a Veterinary Zootechnician (in other words, a Multispecies Doctor). I completed a direct PhD in Science with a Specialization in Neuroscience at the Institute of Cellular Physiology, Biophysics, and Neurosciences of the National Autonomous University of Mexico (UNAM), in collaboration with the Department of Molecular Psychiatry at the Yale University School of Medicine. My work focuses on creating animal models of human diseases. I study the reasons behind behavior, exploring the neurophysiological and neurochemical aspects—Neurobehavior and Neuroethology. I have always been interested in understanding the keys and scientific foundations of leadership, a behavior common in the animal kingdom, including in hominid primates known as humans.  From 2008 to 2021, I was a professor-researcher in the Neurophysiology of Behavior Laboratory at the School of Medicine of the Autonomous University of the State of Mexico.  Since 2021, I have been a professor-researcher in the Neurophysiology of Behavior Laboratory at the Center for Research and Advanced Studies in Animal Health at the Faculty of Veterinary Medicine and Zootechnics of the Autonomous University of the State of Mexico. |
| 15 | Brian J. Zink, MD | University of Michigan Medical School | Professor of Emergency Medicine, Past Associate Dean for Student Programs, Past Senior Associate Dean for Faculty and Faculty Development; Co-Director Medical Student Leadership Development Program |
| 16 | Not provided by the stakeholder | Not provided by the stakeholder | Medical school professor |
| 17 | Eduardo Trujillo Condes | Universidad Autónoma del Estado de México (UAEMéx) – Faculty of Medicine | - Head of the State Department of Teaching and Research, State of Mexico Delegation - Coordinator of Teaching and Research, General Hospital of the ISSSTE in Toluca - President of the Research Ethics Committee, Faculty of Medicine, UAEMéx - Vice President of the Certification Council of General Medicine of the State of Mexico - Full-time Professor-Researcher, Faculty of Medicine, UAEMéx - Leader of the Academic Group: Translational Research and Innovation in Education and Health - President of the Academy of Clinical Integration I, Faculty of Medicine, UAEMéx - Member of the Committee for the Curricular Restructuring of the Medical Surgeon Degree - Verifier for the Mexican Council for the Accreditation of Medical Education - Teaching Coordinator of the Medical Surgeon Degree |
